# SOX7 co-regulates Wnt/β-catenin signaling with Axin-2: both expressed at low levels in breast cancer

**DOI:** 10.1038/srep26136

**Published:** 2016-05-18

**Authors:** Huidi Liu, Emilio Mastriani, Zi-Qiao Yan, Si-Yuan Yin, Zheng Zeng, Hong Wang, Qing-Hai Li, Hong-Yu Liu, Xiaoyu Wang, Hong-Xia Bao, Yu-Jie Zhou, Jun-Jie Kou, Dongsheng Li, Ting Li, Jianrui Liu, Yongfang Liu, Lin Yin, Li Qiu, Liling Gong, Shu-Lin Liu

**Affiliations:** 1 Genomics Research Centre, Harbin Medical University, Harbin, 150081, China; 2 Collage of Pharmacy, Harbin Medical University, Harbin, 150081, China; 3 HMU-UCFM Centre for Infection and Genomics, Harbin, 150081, China; 4 Department of Biochemistry and Molecular Biology, University of Calgary, Calgary, T2N 4N1, Canada; 5 College of Bioinformatics Science and Technology, Harbin Medical University, Harbin, 150086, China; 6 Pathology Department, The First Hospital of Qiqihaer City, Qiqihaer, 161006, China; 7 Department of Microbiology, Immunology and Infectious Diseases, University of Calgary, Calgary, T2N 4N1, Canada

## Abstract

SOX7 as a tumor suppressor belongs to the SOX F gene subfamily and is associated with a variety of human cancers, including breast cancer, but the mechanisms involved are largely unclear. In the current study, we investigated the interactions between SOX7 and AXIN2 in their co-regulation on the Wnt/β-catenin signal pathway, using clinical specimens and microarray gene expression data from the GEO database, for their roles in breast cancer. We compared the expression levels of SOX7 and other co-expressed genes in the Wnt/β-catenin pathway and found that the expression of SOX7, SOX17 and SOX18 was all reduced significantly in the breast cancer tissues compared to normal controls. AXIN2 had the highest co-relativity with SOX7 in the Wnt/β-catenin signaling pathway. Clinicopathological analysis demonstrated that the down-regulated SOX7 was significantly correlated with advanced stages and poorly differentiated breast cancers. Consistent with bioinformatics predictions, SOX7 was correlated positively with AXIN2 and negatively with β-catenin, suggesting that SOX7 and AXIN2 might play important roles as co-regulators through the Wnt-β-catenin pathway in the breast tissue to affect the carcinogenesis process. Our results also showed Smad7 as the target of SOX7 and AXIN2 in controlling breast cancer progression through the Wnt/β-catenin signaling pathway.

Breast cancer is the most common malignant tumor in female and among the leading cancer deaths worldwide, with the incidence rapidly increasing in both American and Asian countries^1^,^2^. Breast cancer classification and stage evaluation have traditionally been based on clinicopathological features (patient age, lymph node invasion, tumor size, histological type, and pathology grade)^3^, but recent technological advances have made it possible to differentiate breast cancers by molecular markers such as ER (Estrogen Receptor), PR (Progesterone Receptor) and HER2 (Human epidermalgrowth factor receptor-2)^4^,^5^. Among the high risk genetic factors hitherto known to contribute to the pathogenesis of breast cancer are BRCA1 (Breast Cancer Type 1 Susceptibility Protein), BRCA2 (Breast Cancer Type 2 Susceptibility Protein) and TP53 (Tumor protein p53), which have high penetrance for the disease^6^. Over the past decade, extensive research has been focused on the identification of cellular signaling pathways involved in breast cancer, such as MAPK (Mitogen-activated protein kinases), PI3K/Akt (Phosphatidylinositol-3-kinase/Akt) and Wnt/β-catenin signal pathways^7^,^8^,^9^,^10^. However, it still seems far away from a full picture of the molecular mechanisms underlying the breast carcinogenesis, which may involve a much greater variety of pathways and factors to be identified.

SOX7 (Sex Determining Region Y-Box 7), together with SOX17 (Sex Determining Region Y-Box 17) and SOX18 (Sex Determining Region Y-Box 18), belongs to the SOX F subfamily. As a well studied developmental regulator in hematopoiesis and cardiogenesis, it has also been implicated in human cancers^11^, e.g., its involvement in suppressing pathways such as Wnt/β-catenin signaling that is highly activated in breast cancer^12^. Increasing lines of evidence have shown that SOX7 may disrupt the transcriptional function of the β-catenin-TCF/LEF (T cell factor/lymphoid enhancer factor) complex and inhibit the activity of Wnt target genes, including cyclin D1, c-Myc and COX-2^13^,^14^. Several recent studies, including our own published work, have demonstrated SOX7 as a tumor suppressor through the Wnt/β-catenin signaling pathway in multiple cancers^13^,^15^,^16^,^17^,^18^. Besides other genes in the SOX F subfamily, SOX7 has been reported to interact with SOX4, which is positively correlated with the status of β-catenin and TCF4^13^,^19^,^20^,^21^,^22^. As an antagonist of SOX4 on Wnt/β-catenin signaling, SOX7 may suppress the pathway through competitive binding to β-catenin^21^,^23^.

AXIN2 (Axis Inhibition Protein 2), an important regulator in the Wnt/β-catenin signaling pathway, is involved in the regulation of cell proliferation, cytometaplasia, migration, apoptosis and other important processes and has been implicated in the development of cancers in the liver, colon, lung, and breast^24^. AXIN2 controls the level of β-catenin in cytoplasm by promoting β-catenin degradation^12^ and forms a negative feedback regulation with Wnt. However, it is not clear whether AXIN2 and SOX7 regulate the Wnt/β-catenin signaling pathway independently or by cooperation.

In this study, we investigated the changes and possible roles of SOX7 in breast cancer by analyzing its expression levels using bioinformatics tools and then experimentally validated the results with clinical tissues. We found that the expression of SOX7 was significantly reduced in breast cancer and this effect was positively correlated with AXIN2. Our results also suggested Smad7 to be the target of SOX7 and AXIN2 in controlling breast cancer progression through the Wnt/β-catenin signaling pathway.

## Results

### Expression levels of SOX7 and other SOX family members in breast cancer and normal tissues

**Figure 1:**
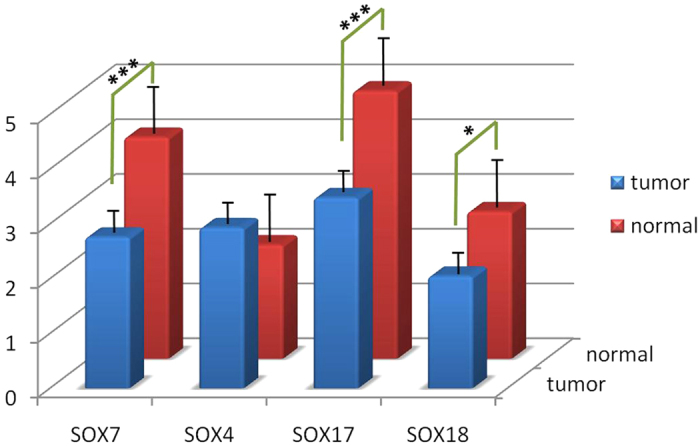
The expression levels of SOX7, SOX4, SOX17 and SOX18 between breast cancers and normal controls in GSE3744. A significant correlation was found between breast cancer and reduced SOX7, SOX17 and SOX18 mRNA levels compared with normal control.

We compared the expression levels of SOX7 and other SOX family members using gene expression profiles by MATLAB (Matrix Laboratory) between forty breast cancers and seven controls in GSE3744, with the FDR (False Discovery Rate) being controlled. We found that the expression levels of SOX7, SOX17 and SOX18 were reduced significantly in breast cancer tissues compared to normal controls (Table 1). In contrast, SOX4 was markedly increased in breast cancer tissues (Fig. 1).

### Profiles of SOX7 expression in different pathological types of breast cancer

**Figure 2:**
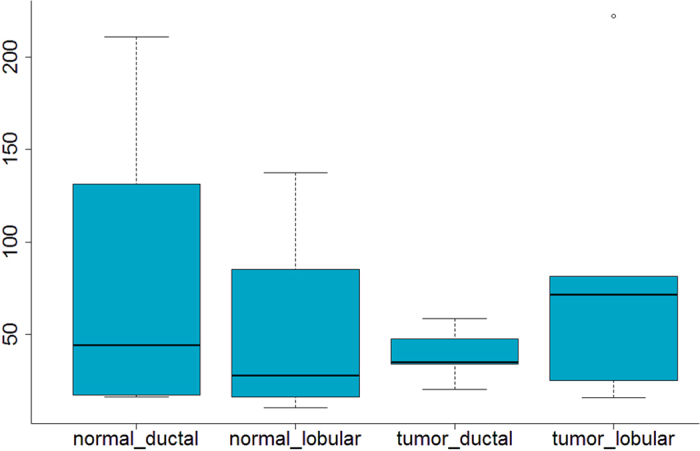
Correlation of reduced SOX7 expression with different pathological types. One-way ANOVA analysis of SOX7 mRNA expression levels among ND, NL, TL and TD. Reduced SOX7 mRNA level was shown in tumor tissue compared with normal control, but no significant correlation was found in the comparisons (*p* = 0.456).

In order to determine whether the relative expression levels of SOX7 might be variable among different pathological types of breast cancer, we performed one-way ANOVA analysis of variance between cancers by R software, involving five invasive lobular tumors (TL) and five ductal tumors (TD), ten normal lobular (NL) and ten normal ductal (ND) tissues derived from the GSE5764 dataset after pre-processing. Variations of SOX7 expression level among the groups were not statistically significant (*p* = 0.456) (Fig. 2).

### Genes co-expressed with SOX7 in the Wnt/β-catenin pathway in breast cancer

Postulating that some genes in the Wnt/β-catenin pathway might be co-expressed with SOX7 and contribute to tumor suppressing, we compared the gene expression profiles between the 40 breast cancer specimens and the seven normal breast tissues. Among the identified genes that were co-expressed with SOX7, we short-listed 3242 by Pearson correlation (FDR < 0.05) in GSE3744 (see Supplementary Table S1) and, using DAVID (the Database for Annotation, Visualization and Integrated Discovery) with a 5% FDR control, we identified 19 genes in the Wnt/β-catenin pathway that were co-regulated with SOX7. Furthermore, when restricting FDR < 0.01, we identified a total of 1470 sets of probes that were co-regulated with SOX7 (Supplementary Table S2). After enrichment on the Wnt pathway, we finalized seven genes that were qualified to the imposed conditions, with the gene of Axis inhibition protein 2 (AXIN2) possessing the highest co-relativity (Table 2). AXIN2, a much studied negative regulator of the canonical Wnt/β-catenin signaling pathway, functions as a tumor suppressor in a number of human cancers^13^,^25^ and presumably plays an important role in the regulation of the stability of β-catenin^12^.

### Expression of SOX7, AXIN2 and β-catenin proteins in clinical tissues

**Figure 3:**
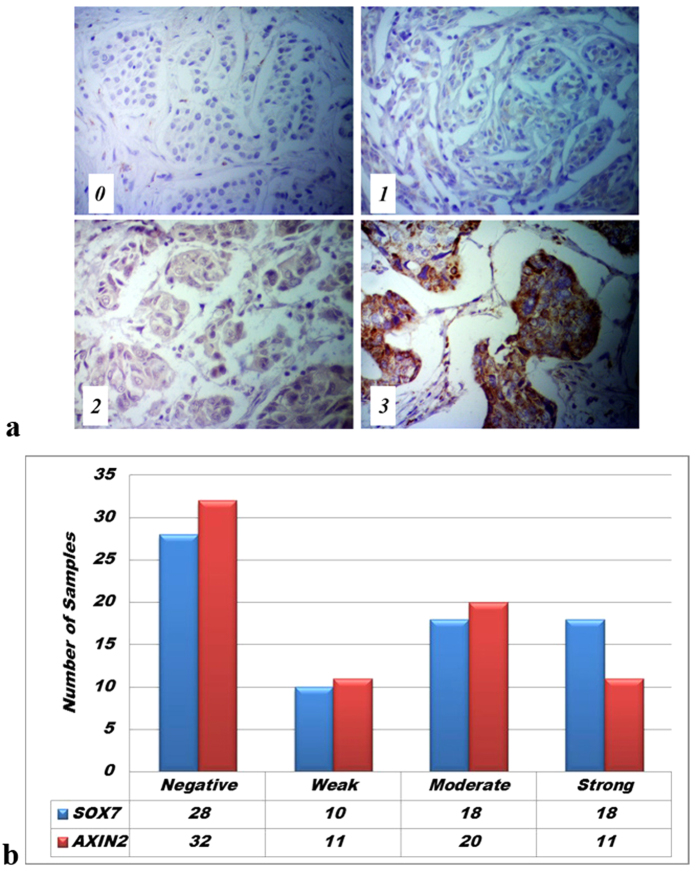
Staining intensity grades for SOX7. (**a)** Examples of 0, 1+, 2+, or 3+ nuclear and partially cytoplasmic staining SOX7 IHC in breast cancer cells. (**b)** Statistical graph was shown SOX7 and AXIN2 after imunohistochemical stains, from left to right, negative (**a**), weak (**b**), moderate(**c**) and strong stain (**d**), over half of samples performed negative or weak expression level in breast cancer. The two genes had a similar expression trend.

**Figure 4:**
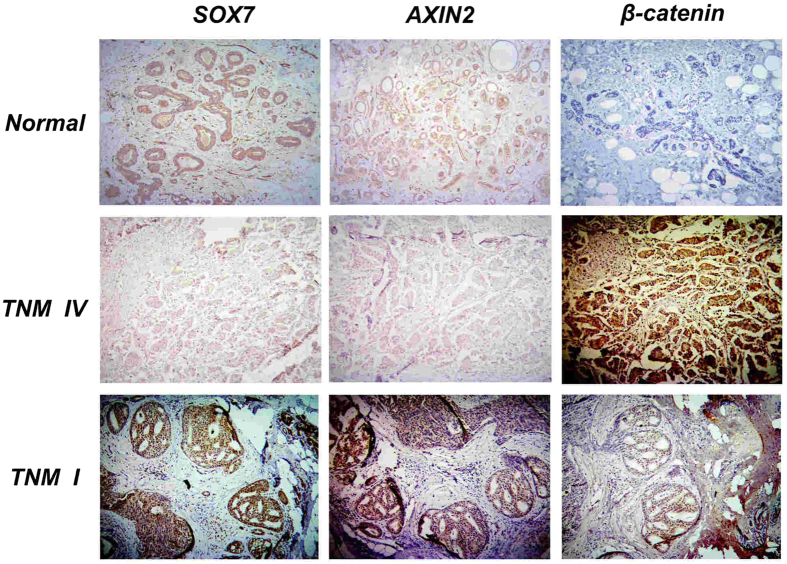
Immunohistochemical staining for SOX7, AXIN2 and β-catenin in normal breast tissues and breast cancer with different TNM stages.

To substantiate the pathway analysis done by bioinformatics methods, we conducted immunohistochemistry (IHC) assays for three closely-related genes in the Wnt pathway, including SOX7, AXIN2 and β-catenin for 30 normal breast tissues and 74 breast cancer specimens with different pathological types, TNM (T: size or direct extent of the primary tumor; N: degree of spread to regional lymph nodes; M: presence of distant metastasis) stages and pathology grades. Student’s *t* test confirmed that all three genes were differentially regulated relative to different TNM stages at levels comparable to the array data. We found that, in normal breast tissues, the staining for both SOX7 and AXIN2 was strong and predominantly nuclear and partially cytoplasmic. However, among the breast cancer tissues, twenty eight exhibited a negative staining (0–+4) and ten had weak staining (+6), with eighteen moderately (+8–+9) and eighteen strongly stained (+12) by the modified Allred score on IHC (Fig. 3). In contrast, expression levels of β-catenin had a reverse trend, i.e., negatively correlated with those of SOX7 and AXIN2 (Fig. 4). Notably, although negative or weak SOX7 and AXIN2 staining intensity was seen in over half of all the breast cancers (38/74 SOX7, 43/74 AXIN2; Fig. 4) and β-catenin also exhibited a weak staining intensity in many cases (54/74 β-catenin Fig. 4), most of the specimens with weak β-catenin staining were from patients in TNM I–II. These results suggest that SOX7 and AXIN2 may function in a similar pattern not only in normal breast tissues but also in breast cancer through the Wnt/β-catenin signaling pathway, particularly at early stages of breast cancer.

### Associations of SOX7 expression with clinicopathological parameters

We analyzed the data for any possible associations of SOX7 protein expression with the clinicopathological features of breast cancers and found, similar to our bioinformatics predictions, there was no significant difference among different pathology types (Table 3). However, the clinicopathological correlation analysis revealed that the down-regulation of SOX7 was significantly associated with advanced stages (III-IV; 22/74, *p* < 0.01). Moreover, the SOX7 protein expression was more frequently detected in the well and moderately differentiated breast cancer than in poorly differentiated breast cancer (G1–G2; 59/74, *p* < 0.01). However, no significant association was found with lymph node metastasis (*p* = 0.310), nor with different age groups (*p* = 0.143). Importantly, SOX7 expression was correlated positively with AXIN2 (r_s_ = 0.379, *p* = 0.001) and negatively with β-catenin (r_s_ = −0.317, *p* = 0.006) and pivotal hub or downstream genes in the Wnt-β-catenin pathway, strongly suggesting a possible role of SOX7 as a negative regulator by down-regulating β-catenin and AXIN2 as a co-regulator through the Wnt-β-catenin pathway in breast cancer. For simultaneous comparison, each type of breast tissues used for photographing was from the same patient (Fig. 4).

### Correlation of SOX7 expression levels with hormone receptors and biomarkers

**Figure 5:**
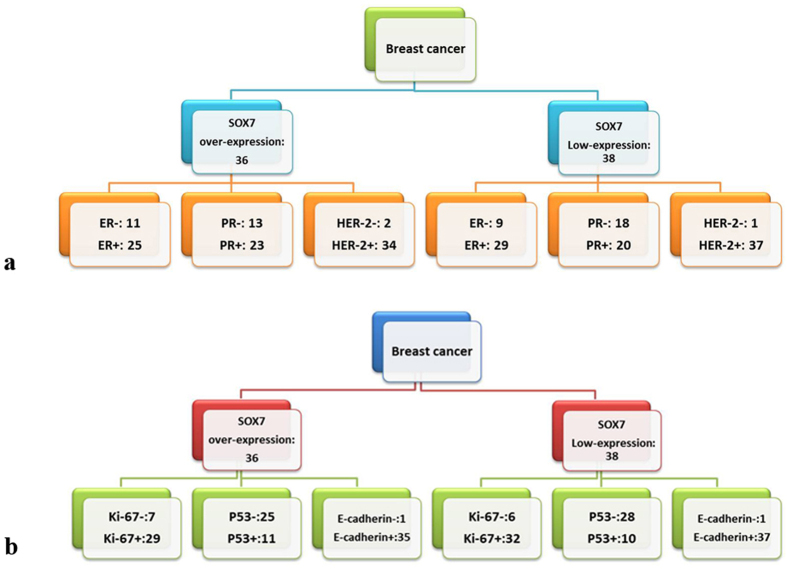
Case Distribution. (**a)** Comparison between expression levels of SOX7 with hormone receptors. (**b)** Comparison between expression levels of SOX7 with different biomarkers.

From clinical data, we profiled the expression levels of hormone receptors for ER, PR and HER-2. After data processing, we found that SOX7 tended to be correlated positively with ER (r_s_ = 0.077, *p* = 0.513) and HER-2 (r_s_ = 0.220, *p* = 0.059) but negatively with PR (r_s_ = −0.090, *p* = 0.438), although the correlations were not statistically significant (Fig. 5a, Table 4). In the same way, we analyzed the correlations between SOX7 and some biomarkers (Ki-67, p53, E-cadherin) routinely used for breast cancer. As shown in Table 5, no correlation was seen between SOX7 and any of the biomarkers analyzed (Fig. 5b). Taken together, these findings suggest that SOX7 might be an independent biomarker in breast cancer.

### Predictions of relationships among SOX7-AXIN2-β-catenin by GeneAnswers

**Figure 6:**
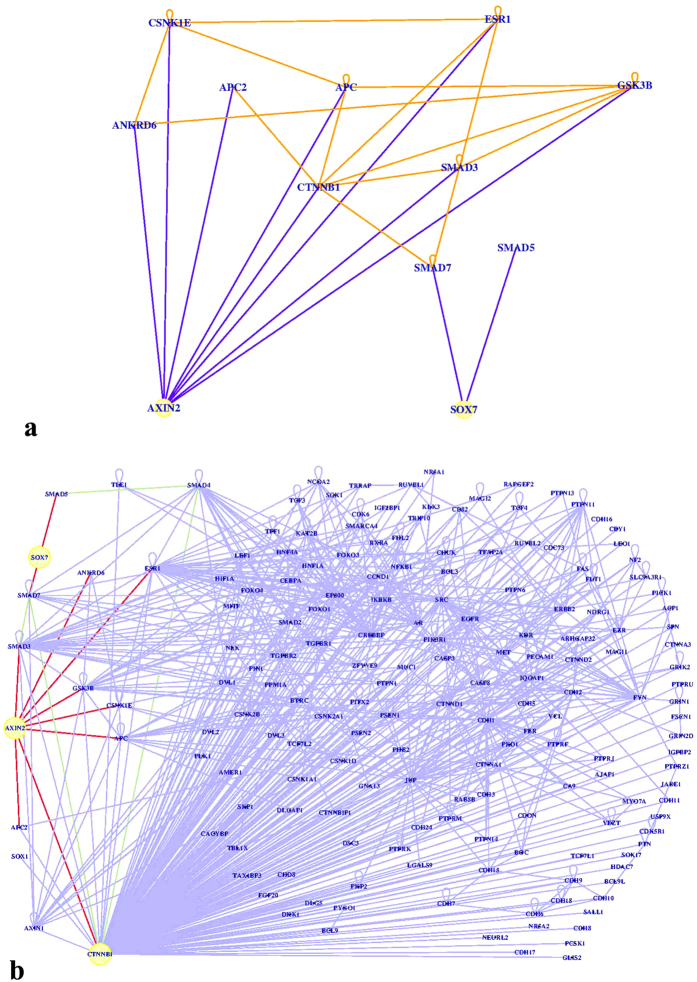
Predictions for the relationship among SOX7-AXIN2-β-catenin by GeneAnswers. (**a)** Axin2/SOX7 relation. The picture shows the relation between Axin2 and Sox7 according to the DOLITE category. The nodes in yellow are the interested genes. The blue links are that directly related to the interested genes, while not the orange ones, the yellow circles indicate that the genes played negative modulatory effects to each other. The picture was obtained with GeneAnswers tool asking for level 1 connection. (**b)** Axin2/Sox7/β-catenin relation. The picture shows the relation between Axin2, Sox7 and β-catenin according to the DOLITE category. The nodes in yellow are the interested genes. The red links are direct ones related to the interested genes. The green links are not-direct ones related to the interested genes. The picture was obtained with GeneAnswers tool asking for level 1 connection.

To understand the mechanisms underlying the functional interplay between SOX7 and AXIN2, we used a “disease pathways” model involving both of them. In the process of mapping a series of genes to functional categories of gene ontology (GO)^26^ annotations using computational tools, many genes may map to several GO terms. To identify disease involvement of genes, one may use disease ontology (DO) annotations^27^. As a gene may be connected to a certain concept of interest (either GO or DO) indirectly via PPI (protein–protein interaction) networks, the PPI network is used to augment the network inference. We thus used GeneAnswers to analyze the data and investigate gene distribution according to the “diseases” as defined by DOLITE (Fig. 6a). The results demonstrated that SOX7 could indirectly regulate CTNNB1 (β-catenin) through Smad7 (SMAD family member 7), which has previously been reported to be involved in the Wnt pathway^28^,^29^,^30^, whereas AXIN2 showed a direct as well as indirect links, via genes like APC2, APC, ESR1, SMAD3 and GSK3B, to β-catenin.

On inspecting the relationships between SOX7 and β-catenin and those between AXIN2 and β-catenin from DOLITE, we obtained similar results indicating that SOX7 displayed direct regulatory effects, whereas AXIN2 affected β-catenin both directly and indirectly (Fig. 6b; red edges are direct links, green ones are not-direct links but relevant for Wnt-β-catenin pathway).

### Predictions for the various roles of SOX family members by GeneAnswers

**Figure 7:**
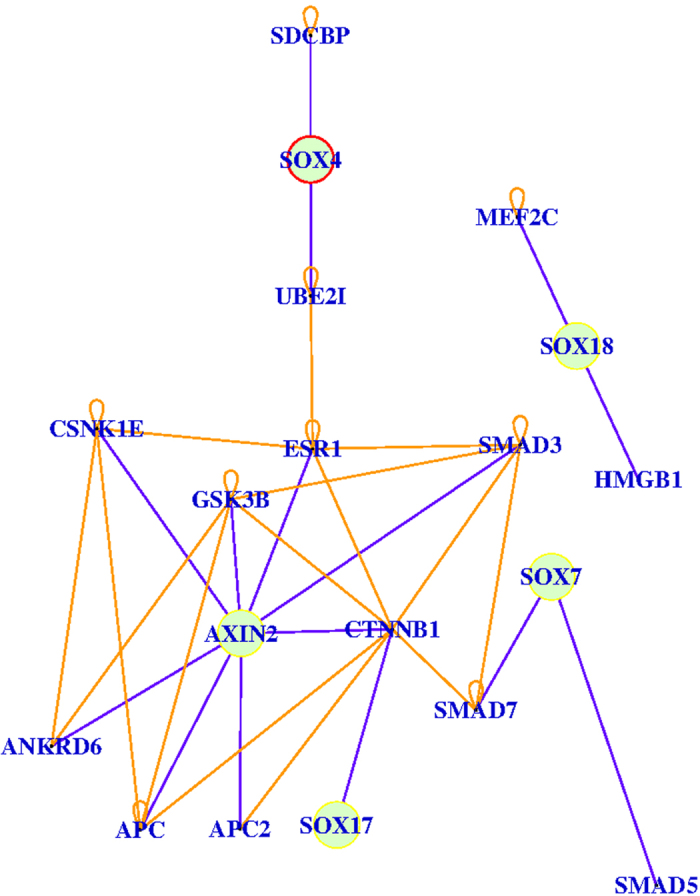
SOX4/SOX7/SOX17/SOX18/AXIN2 relation. The picture shows the relation between SOX4, SOX7, SOX17, SOX18 and AXIN2 according to the DOLITE category. The nodes in green are the interested genes. The blue range edges are direct links, the orange ones are indirect links. The yellow circle represents down-regulation, while the red circle means up-regulation.

In a similar way, we investigated the relationships among SOX4, SOX17 and SOX18 in the disease pathways and found that SOX4 played roles as an up-regulator to β-catenin by a complex pattern via UBE2I and ESR1, whereas SOX7 and SOX17 both were predicted to be down-regulators to β-catenin. As in Fig. 7, SOX17 is shown to have a direct link to β-catenin but SOX18 may have independent roles to the network.

### Analyses by Different Annotation Database Resources

**Figure 8:**
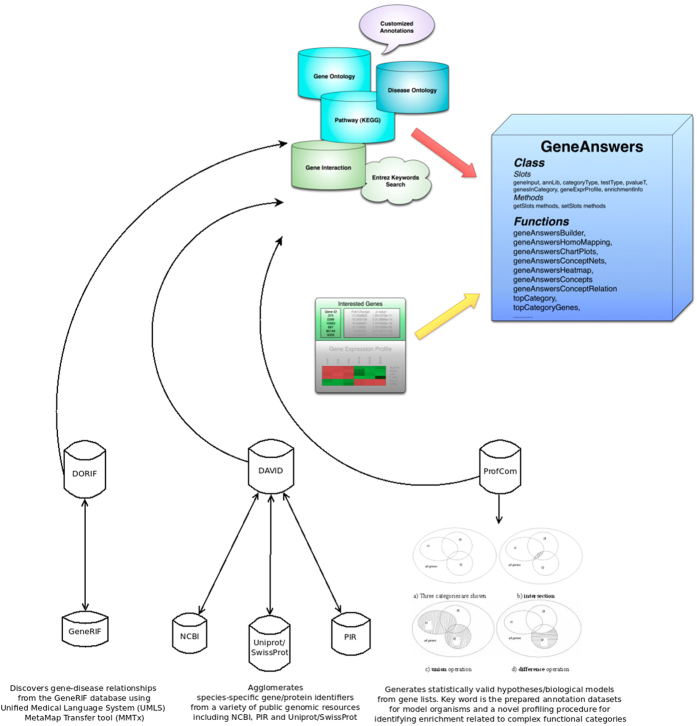
Annotation/Functional DataBases and GeneAnswers. The picture shows the capabilities of each data resource used. It highlights that GeneAnswers is in line with the results produced by DORIF, DAVID and ProfCom.

GeneAnswers solves problems by creating a network of genes-to-concepts and visually highlighting genes that are involved in several functions (Fig. 8)^31^. Furthermore, GeneAnswers tests each concept for its “enrichment” in the gene list versus the genome using the well-defined hypergeometric statistical test^32^. These observations gave us a good level of confidence in the obtained results.

In order to be more confident about the relationships predicted for this set of genes, we performed the analyses using different web resources, including FunDO^33^, which can browse the original mappings referring to Disease Ontology obtained from the GeneRIF database (DORIF, http://projects.bioinformatics.northwestern.edu/do_rif/). Upon querying the DORIF database, we obtained evidence that genes in our list may interact in some way, such as SOX4/SOX17 acting as both antagonists and agonists of beta-catenin/TCF activity (see Supplementary Table S3); this mechanism may regulate Wnt signaling responses in many developmental and disease contexts with aberrant activation of Wnt signaling^21^,^23^,^34^.

## Discussion

Breast cancer remains associated with significant morbidity in female malignancies. It is a huge challenge of current basic and clinical research to seek fresh effective molecular markers for more accurate and efficient use in diagnosis, treatment or prognosis of breast cancer. In this study, we indentified SOX7 and AXIN2 as downregulators in the Wnt-β-catenin signaling pathway. SOX7 has been reported to be a tumor suppressor in many human cancers and its reduced expression frequently correlates with poor prognoses^11^. A previous study showed that SOX7 played an inhibitory role in hepatocarcinogenesis and so might be a novel target for HCC therapy^35^. In a recent study, we for the first time demonstrated that SOX7 behaved as a tumor suppressor through the Wnt/β-catenin signaling pathway in ovarian cancer^15^. Consistently, a latest study in acute myeloid leukemia (AML) also indicated SOX7 to have a negative modulatory effect on the Wnt/beta-catenin pathway^36^. Additionally, several publications have focused on the methylation status of the SOX7 promoter in tumor and normal breast tissues^37^ as well as in MDS patients^38^. Moreover, in endometrial and gastric cancers, the expression of SOX7 and β-catenin is regulated by a negative linear correlation with each other^13^,^16^. Of great interest, SOX7 may have potential usage as an independent prognostic marker in prostate and lung cancers^39^,^40^. In breast cancer, Sui and colleagues for the first time reported that SOX7 is likely regulated by multiple mechanisms and may potentially serve as a prognostic marker for breast cancer^37^. Furthermore, SOX7 has been supposed to be a suppressor to miR-492, which represents a potential onco-miR and participates in breast cancer carcinogenesis^41^. These results all suggest that SOX7 might be a tumor suppressor through the Wnt/beta-catenin pathway in various cancers. Additionally, in this work, we also found the involvement of Smad7 in this pathway to affect SOX7.

To date, little has been known about the possible co-regulation of a carcinogenesis process, e.g., the Wnt/β-catenin signaling pathway, by different suppressors in breast cancer. Findings in this study demonstrate the cooperation of SOX7 and AXIN2 in regulating the Wnt/β-catenin signaling pathway. Firstly, using gene expression files, we found that the expression of SOX7, SOX17 and SOX18 was significantly reduced, whereas SOX4 was markedly increased in breast cancer tissues compared to normal tissues. Analyses by the bioinformatics tool GeneAnswer showed that SOX7 and SOX17 both were down-regulators on β-catenin, while SOX4 was an up-regulator. Secondly, the expression levels of SOX7 showed no significant difference among different pathology types of breast cancer. Of special significance, in our work AXIN2 was shown to have a co-expressing pattern with SOX7, as initially predicted by bioinformatics procedures with GeneAnswer and then validated by immunohistochemistry experiments. Additionally, the analysis of clinicopathological data revealed that the expression levels of SOX7 were significantly correlated with TNM stage and pathology grade but not significantly correlated with hormone receptors or other biomarkers. Thirdly, GeneAnswers was adopted to predict the relationship among SOX7-AXIN2-β-catenin, showing that SOX7 displayed an indirect regulating way, while AXIN2 performed both direct and indirect way to β-catenin. A negative modulatory pattern was shown in both SOX7 and AXIN2 to β-catenin. Between SOX7-β-catenin regulation, Smad7 gene emerged in the process as an essential molecular protein for nuclear accumulation of β-catenin in the canonical Wnt signaling pathway^42^. Our prediction also agreed with previous studies on the way of β-catenin regulation in colorectal cancer^42^ and the process of osteonecrosis pathogenesis^43^. Evidence is accumulating to show that Smad7 interacts with β-catenin and suppresses its degradation in the Wnt signaling pathway in different cancer types^29^,^43^,^44^,^45^. To our knowledge, we for the first time report here the relationships between SOX7 and Smad7 and open the opportunity to elucidate the role of Smad7 in relation to SOX7 or other SOX members in cancers. Based on the findings in this study, we propose that the significantly reduced expression of both SOX7 and AXIN2 may jointly facilitate the pathogenesis of breast cancer, possibly through regulating the β-catenin by not-direct as well as direct ways.

The Wnt/β-catenin signaling pathway is well known to have important roles in various malignancies^13^,^15^,^16^,^17^,^46^, involving a large variety of factors, including SOX7 as indicated in this work. Our work is in general agreement with previous reports on the down-regulation of SOX7 in malignant tissues and in the meantime demonstrated that SOX7 prometed the degradation of β-catenin, at least in part by targeting Smad7. Both their not-direct and direct ways of effects on β-catenin suggest that SOX7 and AXIN2 work jointly as co-regulators of the Wnt/β-catenin signaling pathway.

AXIN2 has been known as a hub regulator of Wnt/β-catenin signaling pathway, playing roles in the tumorigenesis mainly because of gene methylation and mutations^47^. Numerous studies suggest that AXIN2/Wnt forms a negative feedback regulation, in which AXIN2 controls the level of β-catenin in cytoplasm by promoting β-catenin degradation^48^,^49^. Our work reported here showed that SOX7 expression was correlated positively with AXIN2 and negatively with β-catenin, supporting our postulated synergy of SOX7 and AXIN2 to suppress tumor progression by promoting β-catenin degradation^42^.

In conclusion, we demonstrate here, for the first time, that SOX7 plays the role of tumor suppressor on β-catenin by targeting Smad7, together with AXIN2 as a portential co-regulator of the Wnt/β-catenin signaling pathway in controlling breast cancer progression. Clinicopathological data also showed that SOX7 was significantly associated with disease progression and so may be used as an independent predictor of breast cancer.

## Materials and Methods

### Clinical specimens

Clinical specimens were collected from the Department of Pathology, the First Hospital of Qiqihaer City, from January to December 2014. All patients were informed of the purpose of the study and gave written informed consents. All experimental protocols were approved by the Collage of Pharmacy Harbin Medical University Ethics Committee. The methods were carried out in accordance with the 1975 Declaration of Helsinki. The stage and histological grades of all the cases were determined according to the criteria of FIGO. None of the patients received any chemotherapy or radiotherapy before operation. Clinical information of patients was obtained from medical records and pathology reports.

### Immunohistochemistry

The formalin-fixed, paraffin-embedded breast tissue sections (3 μm) were processed for peroxidase (DAB) immunohistochemistry. After deparaffinization and rehydration with xylene and ethanol, endogenous peroxidase was inactivated with a 3% hydrogen peroxide solution followed by heat-induced antigen retrieval for 20 min. After cooling at room temperature, the slides were blocked with normal goat serum working solution and incubated with primary antibodies (SOX7, Santa Cruz #sc-20093; β-catenin, Santa Cruz#sc-7963; AXIN2, Abcam#ab32197) at a dilution of 1:100 at 4 °C overnight. On the following day, peroxidase labeled polymer and substrate-chromogen were applied to visualize the staining of the target proteins. Finally, the specimens were counterstained with hematoxylin.

### Chemicals and antibodies

Peroxidase-labeled rabbit-anti human IgG SOX7 (sc-20093) and mouse-anti human IgG β-catenin (sc-7963) were purchased from Santa Cruz biotechnology. Peroxidase-labeled rabbit-anti human IgG AXIN2 (ab32197) used in immunohistochemistry was purchased from Abcam, UK. Histostain-Plus Kits (SP-9001, SP-9002) and 3,3′-diaminobenzidine tetrahydrochoride Substrate Kit (ZLI-9032) used in immunohistochemistry were purchased from ZSGB Bio, Beijing, China.

### Datasets and Pre-processing

Eighteen sets of normalized microarray gene expression data of breast cancer were downloaded from the Gene Expression Omnibus data repository (GEO, http://www.ncbi.nlm.nih.gov/geo/). The same platform GPL570 was used to detect all the sets. The detailed information was shown in Table 6.

The use of relative expression obviates the need of between-chip normalization, because all direct comparisons between genes occur within individual samples, and inter-chip normalization can preserve order. For this reason, the raw data (.CEL files) for each dataset were processed using the RMA for background adjustment without quantile normalization^50^. Then, each probe-set ID was mapped to Entrez gene ID with the custom CDF file. If multiple probe-sets were mapped to the same gene, the expression value for the gene was summarized as the arithmetic mean of the values of multiple probe-sets.

Probe sets that did not match any known Gene ID or that matched multiple Gene IDs were abandoned. For each of the samples, the expression values of the probe sets matched to the same Entrez Gene ID were averaged as the expression value of that Entrez Gene ID. Genes in GSE3744, the expression of which significantly correlated (Pearson correlation) with that of SOX7, were defined as co-expression genes with SOX7. The pathway information of Wnt/β-catenin was documented in Kyoto Encyclopedia of Genes and Genomes (KEGG) website.

### Standard for evaluation

The stained slides were scored by two experienced pathologists independently, who were blind to the clinical data of the patients. For each tumor specimen tested, a basic and semi-quantitative grading scheme was used to evaluate the immunostaining of its target proteins. The immunoreactivity of the samples was determined by multiplying the intensity of the staining and the percentage of stained area, so the final immunoreactivity score (IRS) was obtained. Staining intensity was graded as follows: 0 (Negative), 1 (Weak), 2 (Moderate) and 3 (Strong). And the percentage scoring of immunoreactive tumor cells was as follows: 0 (0%), 1 (1–10%), 2 (11–50%) and 3 (>50%). Protein expression levels were further analyzed by classifying IRS values: those less than 6 were considered as low expression, while others greater than 8 were treated as high expression. Meanwhile, the scores designated by two pathologists independently were compared. To achieve a consensus score, any discrepant scores were trained through re-evaluating the staining by both pathologists.

### Annotation Database Resources

We used three different annotation/functional database resources to show that GeneAnswers is coherent with all of them. DORIF (Disease Ontology GenRIF) is a data resource validated against the Homayouni gene collection using recall and precision measurements. Its data have been also compared with the widely used Online Mendelian Inheritance in Man (OMIM) annotations. After downloaded the raw data, we have done the apposite queries to highlight the occurrence of the genes whose we are interested.

### Analysis of relation among SOX7 and AXIN2 into Breast Cancer

To engage our analysis about relation among SOX7 and AXIN2 into Breast Cancer, we used R version 3.2.2 (2015-08-14) on Platform x86_64-pc-linux-gnu (64-bit) and running under Ubuntu 14.04.3 LT. Most of the works is done through the Bioconductor version 3.2 and in particular with the support of GeneAnswers v. 1.6.0.

**Figure 9:**
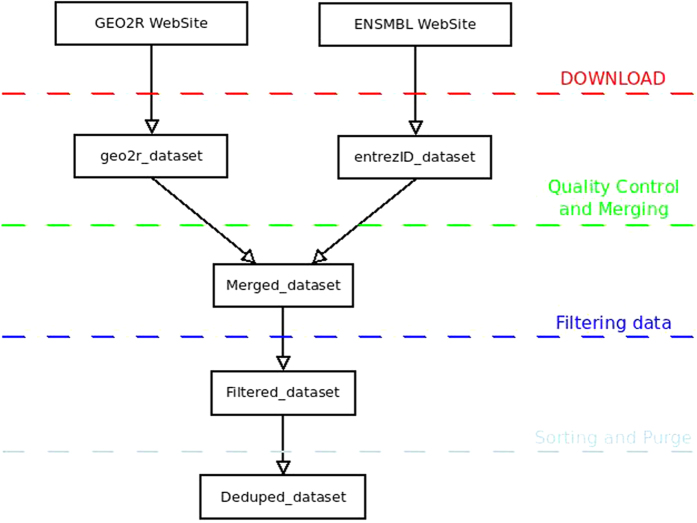
The brief steps done in order to import the data into Biocondutor.

Figure 9 shows briefly the steps done in order to import the data into Biocondutor. First of all, we repeated our investigation using the GEO2R web tool (http://www.ncbi.nlm.nih.gov/geo/) and referring to the GSE3744 dataset, in order to be sure about the starting dataset. The dataset has been normalized and checked about quality using Biobase 2.15.3, GEOquery 2.23.2 and limma 3.10.1 on R. The *“merging action”* was performed using the probe_ID as shared key.

Then we applied a *“filtering action”* dropping all useless information, saving all needed in order to have a better data management and control.

Finally, the *“sorting and purge”* action leads to a dataset sorted by the unique key ENTREZ_ID and purged by all “na” entries.

For purpose of analyzing the imported dataset with Bioconductor, we also needed to execute the followings:

1. Import the Expression Profile GDS2250

2. Apply the log2 transformation with GDS2eSet function, obtaining the eset structure

3. Create the new data-structure composed by the *“eset”* and *“deduped.data”*

Once mapped the geneInput, mapped the genesInCategory, mapped the enrichmentInfo rownames and performed the geneExprProfile, we used the function geneAnswersConceptNet focusing on the pvalue.

In the next, we referred to the function buildNet applied to a single gene, couple of genes and more related genes focusing on the *‘GeneInteraction*’ and *'netMode*’ parameters, with layer set to 1.

This allowed us to look for the *“disease pathway”* where SOX7 and AXIN2 are both involved according to the “diseases” as defined by the lite version of disease ontology (DOLITE).

### Statistical Analysis

The differentially expressed genes of breast cancers vs. normal controls in GSE3744 were calculated by student-t test and the false discovery rate was controlled using the Benjamini–Hochberg procedure^51^. The gene expression level of SOX7 was compared in groups of different malignant states in GSE5764 using one-way analysis of variance (one-way ANOVA). The difference between each pair of the groups was tested by multiple comparison tests and the statistical significance level was given by a student-t test.

For the immunohistochemistry experiment, SPSS version 17.0 was used. Statistical analysis was performed with Fisher’s exact test, chi square test and Spearman’s Rank correlation analysis. Differences with *p* < 0.05 were considered statistically significant.

## Additional Information

**How to cite this article**: Liu, H. *et al.* SOX7 co-regulates Wnt/β-catenin signaling with Axin-2: both expressed at low levels in breast cancer. *Sci. Rep.* **6**, 26136; doi: 10.1038/srep26136 (2016).

## Supplementary Material

Supplementary Table S1-S3

## Figures and Tables

**Table 1:** The expression levels of SOX7, SOX4, SOX17 and SOX18 between breast cancers and normal controls in GSE3744.

Gene	*P*-values	FDR
SOX7	6.9142e-007	1.7642e-005
SOX4	0.0959	0.1365
SOX17	0	0.0002
SOX18	0.0133	0.0418

**Table 2:** The Pearson correlation coefficients between the expression levels SOX7 and downstream genes in Wnt signal pathway list in KEGG with different *P* values.

*P* < 0.001	*P* < 0.05	
**AXIN2**	FBXW11	DVL3
BTRC	RUVBL1	NKD1
CHP	SKP1	PRKACB
NKD1	SOX17	PRKACG
PRKACB	**AXIN2**	PPP2R1A
PRKACG	BTRC	PPP3R2
WNT16	CHP	WNT16
	CAMK2B	WNT3
	DKK2	WNT4
	DKK4	

AXIN2 performs the most notable statistical significance denoted with bold font.

**Table 3:** Correlations of the SOX7 protein expression with the clinicopathological features of breast cancer and Axin2/beta-catenin.

Characteristic	Total	SOX7 expression		*P*
		Over-expression	Low-expression	
All cases	74			
Pathology type				NS
Ductal	63	31	32	
Lobular	7	3	4	
Mucinous	3	1	2	
Other	1	1	0	
TNM stage				<0.01**
Early(I–II)	52	33	19	
Advance(III–IV)	22	3	19	
Pathology grade				<0.01**
G1–G2	59	35	24	
G3	15	1	14	
Lymph node metastasis				NS
+	10	3	7	
−	64	33	31	
Age (years)				NS
<40	5	3	2	
40–60	51	28	23	
>60	18	5	13	
AXIN2				r_s_ = 0.379, p = 0.001**
Over-expression	31	22	9	
Low-expression	43	14	29	
β-catenin				r_s_ = −0.317, p = 0.006**
Over-expression	20	4	16	
Low-expression	54	30	24	

**Table 4:** Comparison between expression levels of SOX7 with hormone receptors.

Genes	Total	SOX7 expression		*P*
		Over-expression	Low-expression	
All cases	74	36	38	
ER				r_s_ = 0.077, p = 0.513
−	20	11	9	
+	54	25	29	
PR				r_s_ = −0.090, p = 0.438
−	31	13	18	
+	43	23	20	
HER-2				r_s_ = 0.220, p = 0.059
−	14	10	4	
+	60	26	34	

**Table 5:** Comparison between expression levels of SOX7 with different biomarkers.

Genes	Total	SOX7 expression		*P*
		Over-expression	Low-expression	
All cases	74	36	38	
Ki-67				r_s_ = 0.048, p = 0.685
−	13	7	6	
+	61	29	32	
P53				r_s_ = −0.047, p = 0.691
−	53	25	28	
+	21	11	10	
E-cadherin				r_s_ = −0.154, p = 0.189
−	5	1	4	
+	69	35	34	
P120				r_s_ = −0.074, p = 0.530
−	3	2	1	
+	71	34	37	

**Table 6:** The detail information of data sets using GPL570 platform.

No.	Type	GEO No.	Sample size	Normal	Disease	References
1	Breast cell	GSE3156	19			Wang Q *et al.* 2011
2	Breast cell	GSE5102	15			Russo PA *et al.*
3	Breast cell	GSE5116	12			Fiche M *et al.*
4	Breast cell	GSE6548	8			Aid M *et al.*
5	Breast cell	GSE7700	8			Hipp JD *et al.*
6	Breast cell	GSE8597	16			Hipp JD *et al.*
7	Breast cell	GSE9086	13			Lu X *et al.*
8	Breast cell	GSE9196	53			Trotman LC *et al.*
9	Breast tumor	GSE3744	47	7	40	Nieselt K*et al.*
10	Breast tumor	GSE3893	24	2	22	Lu X *et al.*
11	Breast tumor	GSE5460	129		129	Wei W *et al.*
12	Breast tumor	GSE5764	30	10	20	Lalleand F *et al.*
13	Breast tumor	GSE6532	741		741	Wirapati P *et al.*
14	Breast tumor	GSE6885	21		21	Saitoh M *et al.*
15	Breast tumor	GSE7904	62	19	43	Lu X *et al.*.
16	Breast tumor	GSE8977	15	7	8	Sullivan A *et al.*
17	Breast tumor	GSE9195	77		77	Wirapati P *et al.* Lallemand F *et al.*
18	Breast tumor	GSE9747	6		6	Schupp M *et al.*
