# Supplementary material for: SOX7 co-regulates Wnt/β-catenin signaling with Axin-2: both expressed at low levels in breast cancer
Source: Sci Rep. 2016 May 18;6:26136. doi: 10.1038/srep26136 (PMC4870566; doi:10.1038/srep26136)
Supplement: Supplementary Table S1-S3 [file srep26136-s1.pdf]

SOX7 co-regulates Wnt/β-catenin signaling with Axin-2: both expressed at low levels in breast cancer

Huidi Liu[1,2,3,7], Emilio Mastriani[1], Zi-Qiao Yan[1], Si-Yuan Yin[1], Zheng Zeng[1], Hong Wang[4], Qing-Hai Li[1], Hong-Yu Liu[5], Xiaoyu Wang[1], Hong-Xia Bao [1], Yu-Jie Zhou[1], Jun-Jie Kou[1,2], Dongsheng Li[1,2], Ting Li[1,2], Jianrui Liu[1 §], Yongfang Liu[1,2], Lin Yin[1,2], Li Qiu[1,2], Liling Gong[1,2], Shu-Lin Liu[1,3,6]*

[1]Genomics Research Centre, Harbin Medical University, Harbin, 150081, China

[2]Collage of Pharmacy, Harbin Medical University, Harbin, 150081, China

[3]HMU-UCFM Centre for Infection and Genomics, Harbin, China

[4]College of Bioinformatics Science and Technology, Harbin Medical University, Harbin, 150086, China

[5]Pathology Department, The First Hospital of Qiqihaer City, Qiqihaer, 161006, China

[6]Department of Microbiology and Infectious Diseases, University of Calgary, Calgary, T2N1N4, Canada

[7] Department of Biochemistry and Molecular Biology, University of Calgary, Calgary, T2N1N4, Canada

[§] Current address: Department of Biomedical Sciences, University of Calgary, Calgrary, T2N1N4, Canada

*Corresponding Author.

Email address: slliu@ucalgary.ca

Supplementary Table S1. Short-listed 3242 genes were co-expressed with SOX7 by Pearson correlation (FDR< 0.05) in GSE3744

PAPOLB	C16orf71	LOC100505942	C15orf60
SCN4A	FLJ21408	C1orf56	DDX25
ASB15	UTS2R	HCN2	LOC730091
LOC100507928	HECW1	LOC728537	CNTN4
WFDC12	HBD	LCN6	PKD2L1
LOC284100	SSTR3	GLE1	GHRHR
LOC730495	HTR6	CXorf36	LOC100289509
PDZD7	ATP4A	IL20RB	PAX2
PRLH	LOC100287024	TFR2	CALML3
NEK10	FBXO36	AMIGO3	FKBP4
CCDC157	NOS1AP	LIAS	ABCA9
TFAP2D	LOC285986	INCENP	TM7SF4
OR5P3	ZSCAN10	LOC100508426	LOC100287627
LOC100131532	LOC285281	CXorf22	TUBGCP2
LOC283868	DBP	WDR64	LOC653786
SLC47A1	CNDP2	LOC100506070	OTOA
NAT8B	LOC100289061	TUBB4Q	GRIA1
CBY3	LENEP	IGHV5-78	MDGA1
NAT8	TTR	CDHR3	FLJ35424
UBXN10	HAR1A	C14orf93	PHF7
C10orf62	KCNC2	ISM2	ACTL7B
SLC6A17	LOC100271832	LOC100509030	TXNDC8
POU5F2	SLC25A25	ANKMY1	TAS2R41
IGSF1	CALCA	LOC100133991	C11orf86
LOC100287576	CSTT	LOC157627	C3P1
TACR3	NCRNA00204B	ACCN4	NODAL
NKX1-1	PLUNC	C7orf69	LOC100289070
FAM166A	CCDC19	LDB3	CHGA
APOA4	HRH2	PDE1B	NCRNA00095
LOC100506319	CDRT15L2	PTHLH	RGAG1
LOC641364	ZFX	LOC100507040	C1QTNF6
CLPS	FLJ40292	TRY6	ZACN
ADAMTS13	C9orf29	TMEM132C	SLC45A1
LOC100129617	LOC100144602	ENG	RHOXF1
LOC253962	CNP	C17orf64	LOC284260
TSPY1	SPTBN4	NAIF1	TMEM88
SOX1	MGC16703	ST6GALNAC4	RNLS
NRL	MYCBPAP	PLA2G12B	GSTTP1
CYP2C9	LOC100287241	ETV1	CNGB3
HNRNPCL1	SUN5	CCDC40	KCNJ15
LOC440563	VIL1	NFS1	PPP1R2P9
LOC649330	TNRC18	GUCA1A	IL1F9

HIGD1B	GJC2	OGDHL	SERPINA7
TIMP4	LOC338667	SPHK2	MPP2
NHLH1	NCR2	LOC100505554	CCDC81
STARD8	MT4	ZCWPW1	FLJ36116
TCN1	KIR2DL1	SLC4A9	TRPA1
C6orf201	IL24	SLC4A4	DHX34
LRRC4B	AFM	GRIK1-AS	NPY5R
CA4	TTC9B	NCRNA00174	ISLR2
CLVS2	PPP5C	SFXN5	SLC5A5
LOC642891	C22orf43	TRIB1	SLC35D3
GRIK3	VEPH1	DDO	CARTPT
ZNF664	NEU2	MAPKAPK2	NCRNA00029
ADIG	C1orf46	PAPPA2	NR4A3
DEFB4A	CDK15	TRPM3	NPHS2
ASB16	LOC100506446	ARHGAP36	LOC90586
LMAN1L	COL4A6	FLT3	12-Sep
ZNF90	TES	DDX56	KLRC3
PRKACG	C7orf51	LOC100287483	SERPINA12
KLHL1	NEFH	LOC100287114	SAMD15
WDR17	CDH22	SPATA4	LOC647310
RRP12	SLC34A3	LOC100288490	SLC16A11
FHAD1	ABCA2	C6orf125	UBE3B
C10orf53	WFDC8	MAPK8IP2	KIAA0895L
C8A	GOLGA8IP	ATP6V0D2	BTF3
MGC23270	ZFYVE28	OSM	C1orf173
PTCRA	GNAT2	LEO1	PER1
FLT3LG	C12orf50	ICA1L	C22orf15
CPLX3	FAM155B	TAL1	NPBWR2
IL20	LOC221122	MEPE	CALHM3
NR2E3	HEXA	FLJ46026	CHST5
OGDH	TMEM159	VWCE	TMSB10
C3orf24	NKX6-3	PCSK1	RG9MTD3
CLN6	TP53TG5	MRGPRX4	SSH1
GNB5	LOC283859	LOC728606	CGB
MYH4	DOC2A	DEFB118	CGB5
NCRNA00118	ZMYND10	C9orf135	CGB7
C9orf44	LOC100505498	KIAA1045	MAPRE3
PA2G4P4	SFTPA1	CC2D2A	GHSR
LOC100130331	CCDC106	ZNF396	OR2H1
AQP2	IL4	LOC100287911	CYP27B1
RAB40A	VPS53	KBTBD10	NCRNA00184
LOC100129895	CACNA1I	RHAG	LOC100128977
DNAJC11	HIPK4	CPAMD8	LOC390940
TMEM151B	C10orf67	PRSS2	ZNF517

DDN	SNAP25	TBXA2R	TMEM144
STAT5B	LOC100130522	LOC100293160	C11orf76
RASGRP4	KCNK10	FAM71E1	TPM3
DNASE1	MYL2	ARID3A	DLEC1
TK2	LOC154092	SLC38A10	HOXA11-AS
YY2	FAM188B	C11orf35	RUNDC2A
MEGF11	ATF7	REPS2	IL1RAPL1
DCLK2	TMC2	SERPIND1	LOC100506797
RPL23AP53	LOC440104	SPACA3	DKFZp761E198
GSX2	RPL28	PLXNA2	EMX2OS
PRIC285	POMGNT1	WDR31	FLJ31356
ZRSR1	LOC100134317	LOC100291232	CTRB2
CROCCP3	LOC284412	PLEKHM1P	PDE12
PPIP5K1	LOC100506856	ATCAY	ARMCX4
GMPPB	KIAA1661	PLCH2	FAM83E
LOC100505515	RGS7	EGR4	ZG16
SPIRE2	RTF1	FAM55C	PDZD3
NEK9	LOC100506002	PRH1	C4orf17
LOC147791	CCDC85B	PRSS3	SLC6A18
NAF1	GNL1	FAM120AOS	CECR2
CCDC140	IQCH	SPAG6	ATP1A1OS
CPA1	C3orf15	LOC100133985	ETS2
MBTPS2	C10orf88	ANKS4B	UCN
ARF3	TEX13A	BAI2	C9orf9
HPS1	SCRN1	PDZK1	ACR
AK1	RUNDC1	CDHR5	LOC339666
TGM4	LOC100128262	NKD1	RFFL
EIF4G2	LOC100130691	NECAB2	GAL3ST3
PVT1	SSFA2	MOV10L1	ZNF527
PAX8	TXLNG	CYP4F2	KCNG3
LOC400927	CHP	HTR3C	LOC100129098
CYP1A2	C9orf131	HAP1	ITGAX
LOC100505820	C9orf144	CCDC85C	ARMC9
PLB1	FLJ41170	AMELY	MYH8
LOC440131	KCNJ5	KIAA1614	C3orf32
LOC100287728	SCGB1C1	LOC100329135	FGD2
VIPR2	ELOVL2	BCL6B	LOC100507198
HBE1	LOC731424	MAT1A	TAAR2
LOC100507164	ACTL6B	NBPF4	PRSS50
AK5	HNRNPM	ZNF331	ANKRD31
C10orf91	PROC	TAS2R39	KIAA1409
C5orf4	WDR76	MBD1	OLA1
FBXO39	FRMD8	CD300LG	BRPF3
FLT4	AMPD3	SSTR5	CACNA2D4

COASY	MRAP	FNDC8	KATNAL2
MEGF10	KIRREL2	MGC50722	RSPH3
TRAF7	TTC26	GAD2	HNRNPA1L2
LOC283761	SYT3	DCDC2B	CES1P1
NUDT10	EML2	CD24	PAH
DUSP26	TPP1	LAMC3	RABGGTA
FAM22F	RNF122	GLOD5	PTPRT
SLC2A9	SP5	BAI1	CACNA2D3
ATP2A2	COX4I2	CCR10	RDH8
RPS27	LRRN3	WNT16	C20orf181
RIMBP3	GNAT3	ONECUT1	VHL
RIMBP3B	TSPAN16	SLC22A9	MPV17L
RIMBP3C	LRP1B	ZNF445	TAPBP
CPB1	HUWE1	LRFN1	LOC646168
EIF4EBP2	SLC30A3	THOP1	LOC100131733
SCG3	DNASE2B	ANKRD29	TMEM201
ZNF79	LOC100509661	FSD2	GAMT
CHRM2	OVOL3	CPNE4	SORCS2
GAPVD1	ARHGEF15	DSCR4	ADAD1
FAM55D	ASMT	LASS1	MUC17
POLD2	MBOAT7	C17orf88	TNFSF9
FAM24A	LOC100132207	PPP6R1	LCN10
TPD52L3	ABO	ART1	SCN3B
PEAR1	HNRNPA1P10	FAM41AY1	C5AR1
ADRB3	CYGB	FAM41AY2	LOC729683
C1orf27	SP8	C1orf91	BPIL2
ZNF490	MPPED1	LOC151171	GPER
SCARF1	GATC	GRN	UBE2O
SAMD14	SLC5A10	CCIN	ZNF174
ANKRD23	AGFG2	CD34	DSPP
RDM1	WASH5P	FAM162B	ZNF17
PIGG	HGC6.3	INSL3	PLAC4
ZBTB16	HEATR4	PPP1R1C	ATP6V0A1
ATOH8	LOC153682	ATP9B	C18orf25
CDHR2	LOC441601	CRH	ZNF598
CIB3	MBL1P	ADAMTS7	ZNF843
TSGA13	TPH1	CCL15	WDTC1
TMEM221	ENOX1	UGP2	GLS2
C20orf166	C7orf63	TSFM	AAGAB
LOC100133445	DNAJA4	LAGE3	CCDC96
PPM1N	C16orf74	LOC100287704	SCLY
CELA3B	C9orf103	HNRNPC	CYorf15B
ZFY	UQCC	LOC79999	FOLH1B
DGCR11	TAS2R38	RDH5	FLJ11292

KIF24	CRYBB1	WDR78	CHGB
FLRT1	LOC100132062	ZNF736	NCOA5
PROCR	LOC100133161	CALM3	AIRE
ITPK1	LOC731275	KCNIP3	TOLLIP
LOC340094	LOC100129268	SLC12A9	LY6G6F
RGS12	RFX2	TRIM66	FLYWCH1
LOC400768	SEC14L2	ASB14	NCRNA00260
BRI3BP	ASTN2	C17orf68	OPN1LW
11-Mar	TBC1D28	GARNL3	OPN1MW
LOC100507250	DYDC2	LOC100133669	OPN1MW2
NR1H3	TOR1A	ZNF324B	ANKRD2
SLC2A8	SLC6A13	CH25H	SIX5
NRSN1	C12orf65	HBEGF	SCN8A
RNF157	ACP5	C14orf104	TESC
ACTR3C	LOC100133308	NHLH2	ZER1
KCNC4	SDSL	NFE2L2	SUV420H2
TM9SF4	LOC339807	KCND3	SPATA2
FAM76A	CNTD2	RTN1	SMC1A
PRO2964	C6orf165	RUNDC3A	LOC100132078
CAV3	SALL4	NNT	NFASC
DEFA6	IGSF22	ZNF781	LOC643714
CNR1	FAM122A	ADPRHL1	DYSFIP1
EPHX2	ADAMTSL4	NOX1	LRRC33
GNS	LOC283174	GMPR	CYMP
C19orf30	LOC220980	LOC100508853	WDR1
LOC100507066	PI4K2A	SLC22A8	COMMD4
LOC100507384	LOC100233209	C16orf92	RCE1
MED13L	LOC100133920	CSPP1	CACNB2
SLC9A3	ZNF135	DYRK3	SERTAD2
LOC1720	LOC100287813	ADD1	PHRF1
FBXL18	SEMA6D	KANK3	LOC339166
REM2	LOC100505470	LPIN3	LOC284939
LOC728012	LOC646576	SULT2B1	MMD2
NXNL2	RADIL	SLC26A3	SNX27
MYH15	FCRLB	PDE4C	STAC3
RBP2	SLC27A5	LOC100507408	NOL9
HNRPA1P5	ZNF81	PADI3	SULT4A1
LOC642817	TMEM120B	NLGN4Y	KCNK17
TLN2	GALNT5	RTN4IP1	BMP8A
FOXD2	UBQLN3	DPF1	SNED1
VPS18	LENG9	FAM182A	GPX8
TEX11	OR51M1	FZR1	MTHFR
LOC100128416	HPS4	ZC3H12A	DKFZp566F0947
ARHGAP6	APOOL	KLHL25	ZNF219

C7orf50	DUX2	SCAMP5	RASSF9
FUZ	SRSF4	SARDH	NKX2-8
LOC100509011	NCAM2	GOLGA6L4	EPO
PRSS47	DUX4	LTB4R	FLJ32955
GGT7	DUX4L2	POU3F1	PRMT8
C2orf81	DUX4L3	FLJ14107	LOC100129461
PA2G4	DUX4L4	L1TD1	FGF10
TRPM5	DUX4L5	PRG4	LOC283484
RBBP8	DUX4L6	DUOXA1	SDK2
LCE1E	DUX4L7	LOC339400	GPR62
AP4S1	C9orf79	LOC283403	PTGIR
MUC3A	NR6A1	MERTK	MNS1
DUSP1	TSGA14	C4orf23	DPPA3
ADAMTS4	NNAT	SPN	TEX101
ADCY1	MGC14436	SRC	LOC100129726
SREBF1	SFTPB	LOC100130264	LOC100506553
TSPYL1	CAPN11	FAM165B	SYTL5
FAM102A	DDX3X	GLTSCR1	ABR
HIVEP3	LOC100287556	EGR2	LOC253805
ABCB9	RECQL5	MYOC	LRFN3
LOC387723	STAB2	LOC100505555	HIST1H1T
FOXJ1	C20orf132	TLR5	ACSM3
LOC222159	C1orf55	FLJ12334	BASE
LOC100288123	HACE1	PRB1	DHDDS
TEK	OTOR	EGR3	PPIAL4A
C19orf29	GCNT4	CCT6B	LCN8
LRRC46	FCN2	C20orf26	LOC613266
C11orf72	ACP2	NTS	EPDR1
SCN9A	RNF208	CATSPER2P1	THTPA
TUBG1	ENPP7	HYAL3	OPCML
LOC642980	G6PC3	CASC4	LOC445341
FLJ32063	LOC100507255	ZNF528	HCG27
VAV2	CXorf64	C12orf42	GOLGA6L1
LOC285638	SEMA4A	CPNE6	GOLGA6L6
LOC415056	DCAF11	MED25	HSFX1
ARL17B	EHD4	ZSWIM5	HSFX2
LOC100130654	NDUFA10	LOC100506080	NECAB3
GAD1	ADAMTS10	KIF9	WRAP53
TSNAXIP1	EHBP1L1	PNMAL2	SETDB1
FCGR2A	PAX4	FASN	ANKFY1
RRAGB	PMS1	CLIP3	AOC2
SIK3	ZNF254	GRINA	LOC100129716
KIR2DS1	GLRB	MTOR	C16orf81
PIGO	FAM154A	PARD3B	CELF5

SEPN1	C18orf2	ZNF556	C4orf43
LOC100507371	FLJ40288	NCRNA00167	LOC253044
CASP2	SHE	ERGIC3	SKA1
PRDM14	LOC389765	NCRNA00265	MYO3B
SDHB	FOXRED2	DDX53	KCNRG
XDH	LOC145837	PROM2	SPINK13
LOC100130938	FKSG2	CAMK2N2	ZC3HAV1L
KLHL22	DBF4B	SNORD8	SEMA5B
SIRT3	UNC93A	EIF2AK4	B4GALNT2
RAB40AL	PSME3	RLTPR	C11orf65
SYK	PQLC2	ATE1	ZNF846
SOAT2	HS3ST6	DEFA5	MAP3K12
APOB48R	SPATA18	SPG11	HOXD9
LILRA1	LOC100130856	TOR2A	LOC644714
TPPP	GRIN3A	FAM82A2	TRIM25
NUP210	HLCS	PRPH2	ADC
LOC100129503	TMCO5B	TMEM175	NPY1R
RAD21L1	SRRT	FOSB	SP2
PTGES	C12orf34	EVI5	LONRF2
C10orf112	TCTN2	COL13A1	HOXA4
C21orf84	LOC644090	LOC729652	ADM2
PMCHL2	TMEM136	XGPY2	UTP15
LOC284385	SPRR2G	FAM46B	PHF21B
LOC100132726	NCL	LOC339988	CHM
LOC100506819	LOC100505644	SYN2	LOC728099
GPR144	BTRC	RDH13	C21orf74
IGSF9B	ROBO4	LOC100507454	SYCE1
KIR2DS2	ANKRD39	LOC100289333	APOL5
ATRIP	PRCD	GGN	ZNF385A
GOLGA6B	SPAM1	ZNF585A	FBXO15
GOLGA6D	IPO4	CCDC57	LOC100134445
CD33	CCDC103	PLEKHM1	PTGER1
AMAC1	C1orf49	HTR1B	HTR2C
SV2A	WEE1	OPTC	RUNDC2C
FREM1	PRPF19	ARPP19	LOC286154
ZC3H3	HSPA4	SNORA43	PEX19
LOC100287590	PRDX3	INHBC	ASCC3
C11orf36	MAGEB3	ASAP1	LOC100287529
SERPINB13	FNDC1	OVOL1	LOC388889
DMBX1	C4orf37	CHIA	TSPYL6
LOC284788	LOC84931	UPK3B	RPS14
ZNF876P	SLC5A8	C7orf53	PDIA2
PRKACB	TCTN1	FAM120A	FAM100A
RGS5	MGC45922	KCNH6	DKFZP434L187

SLC22A11	SERPINA4	LOC100132273	KLK13
LRRC6	NOLC1	LOC100510391	C17orf53
FAT4	SPAG8	HFE	LOC100505711
KLHL4	SEMA3F	MAPK13	C5orf45
LOC100287045	CST11	RABL3	KCNA4
OR2B6	UNQ6975	SEZ6L	AHCYL2
ZNF75D	CDX1	MAP6	PRR12
RIPPLY2	FLJ35934	LOC646982	C10orf103
OPA3	SIGLEC6	HDAC8	LOC339593
SLC18A2	ESR1	TTBK1	LOC100288985
AGTR1	LOC284551	C17orf55	KIF12
C1orf84	BAHCC1	ROBO2	SIAH2
PAK7	RFX6	LOC283788	TREML1
ADRBK1	XAGE3	ULBP3	GPRIN3
CPT2	SFTPD	KCNT1	THBD
KCNJ9	FSIP1	MS4A5	CD68
THAP6	LOC286442	DNAH3	TTTY5
FAM167B	PIGQ	LOC286297	YSK4
TRPM4	ANP32C	MOBP	KCNJ6
CXorf27	PSMD4	ALDH16A1	ZNF597
DOCK1	TDP1	LOC26172	APOM
NCOR2	LOC100131642	PTGER3	ALDOB
VPS26B	LOC100506267	LOC390705	LOC400541
LOC388387	C1orf105	LOC100506880	CD80
KRT31	LOC100507443	PYGO2	SLC37A2
TRAF3IP1	FAM49A	MYH16	ZNF366
KCTD17	HIPK2	OK/SW-CL.36	FRMPD4
NUDCD3	LOC100129463	TUBGCP4	IL1F7
BSDC1	OR2C1	MEGF8	SHROOM1
NADK	KRTAP5-9	LOC100216545	LOC100499221
SPACA1	NAALADL1	LOC100289045	POFUT2
LOC100509093	NUP62CL	MANEAL	TRIM9
NCSTN	LOC100286999	UCN2	PRUNE
RIN1	FAM116B	HBB	CAMK1
ZNF808	MFAP3	ZGLP1	WWOX
FAM160A1	GLI4	ACSM2B	DSTNP2
MST1P2	ELAVL4	PDIA3	CSE1L
BTD	GPR120	CBLN3	TMX4
SEZ6L2	PARP10	SLC43A1	LGALS3BP
ZNF589	GAS2L2	CHRNB2	CALML6
LOC100507816	APOC3	C4orf6	AXIN2
LOC286254	GFRA4	SNRPF	LOC100506848
TREML2P1	GPIHBP1	PRRG2	GTF2A2
DOCK5	ASPG	ARHGEF11	ULBP1

LOC100506728	AIP	KDM4B	ZNF425
PHF20L1	VCX2	GLI1	DDX54
KIAA1239	LOC100169752	MST1P9	LOC100507111
TMEM150A	OR1G1	ANXA10	SNORA74A
AGXT	C1orf89	GTF2IRD2	HPYR1
NAPSA	CDNF	HAGH	C19orf6
SCN2B	PIH1D1	CIITA	MFN2
ALPPL2	MPZL2	LOC100506649	LOC400590
LOC440602	CCDC117	LOC100508278	LOC284648
FAM138A	C15orf17	ZNF789	CHST14
FAM138B	MYOZ3	LETM1	CASP10
FAM138C	HAUS1	NRXN3	ACSS3
FAM138E	ATP6AP1	PLEKHA7	RAB5C
FAM138F	LOC283070	BPIL3	CRTAP
LOC152578	UNC45B	C7orf52	STC1
LOC100506677	OR9A1P	CYP24A1	C12orf54
C6orf1	TSSK2	LOC100287037	9-Mar
C19orf57	GYPA	MAGED4	FOXP3
RASGRF2	PPM1M	MAGED4B	LOC100132356
MOCS1	DCDC5	C1orf183	CADM4
PIGT	MTERFD3	GSS	LOC255411
LOC728804	KLRC1	SIGLEC1	MLX
IQCF5	KLRC2	VDAC1	IL17F
LOC400238	MAG	GRK7	LOC93463
ELFN2	TTPAL	LOC440330	TRAF2
C9orf98	TSSK4	SRRM3	ADCY9
GFM2	WHSC1	LOC100289508	MAGEE1
HIBADH	NDUFB4	AKNAD1	SPATA5
FAT1	ZSCAN22	OR2W3	KRTAP2-4
LOC100287482	WDHD1	CBL	PPIA
FLOT1	LOC440934	SPPL2B	LHX5
INPP5A	LOC728819	MSL3	PRL
IGSF8	ADAR	DNAH7	RAB11FIP3
TRANK1	FKRP	HOXD1	EPS15L1
CABLES2	RFX7	C1orf43	C10orf31
ATPAF1	TMBIM6	GIPC3	ANXA3
IMPDH2	PRAMEF10	AMPH	ZNF236
LOC100129722	NDST1	DIO3	MRPS27
NTRK2	LOC100506476	TMEM145	TTC25
ZNF655	AQP1	HNRPDL	URM1
PLK3	NCKIPSD	GOLGA7B	TMCO5A
FMO6P	KLK4	FLJ13439	C10orf27
UBTF	KIAA1609	ARSB	LRRC16B
CDR1	MYL10	C6	AIM1L

LOC100288656	C20orf203	LOC100130815	HLX
TUFT1	UNC13C	RNF149	IQCA1
ICAM4	AIM1	LOC100131053	DTX3
PDE4A	CAPN13	IMPG2	GALNT3
FBXL21	TBRG4	FLJ31713	KALRN
FNTB	LOC338651	SLC25A41	NPPA
LSM14B	C1orf110	C1orf66	PSMC3IP
OK/SW-CL.58	PATE1	LOC283854	SERPINA1
LOC729224	C10orf58	LOC100132319	RS1
MCCC2	C3orf42	SLC25A34	TBC1D15
HRAS	DNAJC5G	LOC100287426	HBA1
S100A4	ITGB1BP3	C5	HBA2
SOX3	LOC388780	KIAA1737	LOC100132570
TYR	NSUN4	RAD50	GPD1
LOC339468	UCP3	NCRNA00173	FAM198B
TRPC4	FGD3	VSX1	CFHR2
NENF	MC2R	HS6ST1	ESYT1
IL20RA	DSCAM	CNNM2	FAM71B
GAS8	LOC100506014	CCDC90B	GPM6A
FAM125B	KRT82	CARD9	FBXW11
KIR2DL5B	C11orf16	AOAH	FMN1
KIR3DP1	ZNF446	CCDC37	CCL17
FNDC5	ACOT11	MAL	YTHDC2
LELP1	SAMD10	LOC100499193	C14orf102
ZNF14	GRM4	CPEB2	GKAP1
PXDN	LOC100505875	MYO7B	PMP2
DCAKD	SMTNL2	CD97	NEU3
DDAH1	KLHDC9	PLEKHG4B	ODF3L1
MAOA	KRTAP4-4	KIAA0090	KCTD1
FAM169B	OR1A2	ZBTB7B	DAZ1
CDK4	FOLR2	MOBKL2C	DAZ2
MUC4	KIAA1804	IFT122	DAZ3
GPR135	ATF6B	EGFL8	DAZ4
MRRF	C3orf78	TMEM229B	ZNF865
ARMC7	MKL2	OAS3	TAF13
LOC100509279	DHX58	XRCC1	ASB12
RHOBTB3	KIRREL3	DRP2	EPN1
RUNDC2B	HNRNPU	ZNFX1	ARSF
RPRD2	LOC100507374	C11orf21	LOC100144603
PLEKHA5	BAIAP2	CD177	PRSS35
OR2L2	GREB1	LOC100506858	RPGR
HMGXB3	TBX1	LOC400573	KCNF1
SLC48A1	OR52D1	LATS2	FAM32A
NTF3	INSL4	DIO1	SLC45A3

ZNF554	LOC100506165	BCAS3	C5orf46
WDR73	GRPR	EPS8L3	KLF1
PRH2	NRG4	UBE2V1	ASPHD1
TCTEX1D2	MID1IP1	FERMT2	DEFB108B
PECR	CECR1	ADRA1B	DEFB108P1
AQP12A	CECR6	LSM3	NAG18
AQP12B	CORO7	SMPD1	C3orf22
LOC401320	TBC1D13	EXOSC6	DUSP8
LOC400940	C18orf16	CBLN4	CYP4F22
CC2D1B	DDX60	METT5D1	MDM2
LOC553103	C14orf45	KRT78	FLJ32756
ZNF316	RDH16	CYSLTR2	LIMS1
LOC100288224	ADPGK	MSTO2P	NGB
TMCO7	C8orf51	MRI1	FLJ31945
KRTAP19-3	TEDDM1	RPS23	HLA-J
HCG18	C4orf49	DKK4	PTPRK
KLF8	TOR1AIP2	LY6E	SLC9A1
FLJ37798	C6orf97	C1orf94	CS
LRDD	C2CD4B	UBE3C	CNTN6
S100A6	LOC100288797	ELP4	PLVAP
C1orf226	MAPKAPK5	GJA8	ACY3
ABCD1	TERF2	SLC6A11	PTH1R
GORAB	LOC151300	MGC12916	GPC3
TOM1L2	IDS	LOC100288104	C2orf83
CTNNA2	JRKL	FOS	FITM2
CITED1	VTCN1	LOC286359	GATS
RUSC1	PDSS2	RORC	CEP250
TMC8	MYLK3	PNPLA1	LOC100287587
DKK2	ARIH2	PRTN3	TMC7
SEMA3G	RTEL1	ZNF705G	C1QTNF7
LOC729178	PCDHB11	THAP4	RPL23AP7
ATP5D	HTR7	ATXN2L	KLHL32
PGGT1B	TRAPPC9	NES	ZNF469
CCNC	CAMKK1	PEG3-AS	NHLRC4
GTF2IRD2B	DVL3	C21orf104	PRUNE2
CASS4	STON1-GTF2A1L	NUP205	MUC13
RFX5	NDUFS7	RRN3P2	NCRNA00290
BAZ1B	LOC339352	POU6F1	LOC100131496
CPN2	LOC642826	LOC646482	SCN7A
FBXO44	C16orf73	YAF2	CSH2
HP	C8orf56	INSIG2	ARSG
ANGPTL6	HHLA1	C15orf24	ZNF609
DCUN1D3	KLK12	LRRC29	ACCN1
RSL1D1	LOC100287407	SIGLEC7	NCRNA00202

LOC100133131	TMPRSS12	TM9SF1	C21orf96
CCNB3	TRNT1	AGPAT6	LOC145694
NCRNA00112	OR10D3	NKAIN3	KIAA0408
LOC100506128	ACOX3	KIAA0947	TMPRSS11B
LOC100509355	POLR3E	KCNJ3	KCNS2
EDC3	KIF2A	FKBP15	FFAR1
TUBA3C	FLJ44606	AKAP5	CCDC21
CDC40	MEAF6	SEMA4D	ANK3
BTBD9	FTO	RALGAPB	BAT2L1
MGC39372	PCP2	KCNK3	WDR93
GEMIN7	PBX4	EDAR	UBE2K
TMEM25	UBAP2L	IRGQ	AKT1S1
FAM167A	LOC100506571	DAGLA	HLA-DOA
LOC100288099	CDKN2AIPNL	MAP3K1	IFNA6
SMG7	CBFA2T2	CTNNA1	LOC100509139
CARD14	ATF3	PNPO	C7orf60
OR3A2	NCRNA00277	MGC44328	NMNAT3
LOC100128946	CSRP2	GBP6	TTC39C
IQSEC3	DCDC2	CACNA1G	OR1C1
CP	FAM71A	NOTCH4	CLEC4C
CASP14	C1orf83	LOC400655	LOC100505976
LOC144817	TIMELESS	SRRD	C17orf79
LOC100508869	BEST1	CCDC148	KIAA0319L
ADAM20	SLC30A1	SLC25A40	FAM19A5
TAB1	TNFRSF18	LOC654841	LOC100509655
ELP2	HIST1H4F	SLITRK1	CDC42SE1
WDR55	LOC100130353	FUCA1	C18orf32
DPH3P1	PIGP	ACRC	MYH13
C2orf18	LOC100288810	VWA3A	HNRNPK
PLP1	COX6A2	DYM	AKR7A3
LOC645744	MAP6D1	PSEN2	MUC3B
ABCC5	RNF123	DHPS	LOC100289312
C22orf27	KIF27	RPS6KL1	PSORS1C2
RET	CDRT1	LOC646903	POU5F1P3
C14orf49	ALOX12B	KCNA6	KPNA6
LYZL1	PAGE5	GPR162	SUMF2
LYZL2	KIAA0141	IQSEC2	SPATA5L1
STX17	MRPL42	MECR	PI4KB
DUSP2	PEBP1	LOC100289641	CYP8B1
NOVA1	TMEM106A	KRTAP10-11	CKMT1A
THEM4	FBXW2	TRIM58	CKMT1B
GUSB	SNORA66	ECM1	HNF1B
C1orf118	TRIM65	KCNQ1DN	H2AFY
PSME1	IGSF10	TMEM155	LOC100128281

LOC100509212	BDH2	ZADH2	TRBV7-8
MRO	C1orf95	DSEL	LOC100288673
CCDC26	SUGT1P1	TSHR	PNPLA4
INPP5E	BCAP31	AIFM3	TMSB4Y
CAMK2B	REC8	CXorf24	SKP1
GSDMD	LOC100507620	CCL4	KLF14
NR4A1	LOC100289610	CREB3L2	DHRS4
EPHA10	C15orf55	C14orf169	DHRS4L2
WDR90	ZCCHC4	DIRC3	C6orf203
KRT35	GNG3	ARHGAP1	KRTAP3-3
TRIM67	CD55	ATP6V0C	C11orf75
MYL5	PCDH10	ADAM28	PCDHB18
LOC285735	H2AFV	LOC100506436	C10orf85
CSNK1A1P1	FLJ42418	CCNF	LMOD3
MPV17	PIGX	PRKAR1A	LOC100240726
DSCR10	CARD10	FJX1	OMG
LOC100287216	NAP1L4	MID2	TBC1D2B
IRS1	LOC100508409	SYAP1	GPBAR1
C16orf79	LOC100130232	TCP11L2	LOC340357
C8orf34	TBKBP1	LOC255654	TRPC5
FCN1	PRLHR	P2RY1	DNALI1
APOBEC3F	CDC42BPG	FBXW4	ASAH2B
HSD17B4	C4orf21	LOC731789	STRA6
FAM27A	HTR1E	PRMT7	LOC646808
FAM27B	ANKAR	MAP2K5	C19orf66
FAM27C	BEGAIN	COMMD10	C8orf73
C22orf31	CCL22	FAM13AOS	HIP1
MRPS2	LIG3	TNRC6C	PF4V1
CCDC61	LOC285847	AKT2	RAB11FIP4
BCL2	SEC14L3	C9orf47	HSPB1
GLIS2	QRSL1	RNF207	SRM
ANKRD36	PHLPP1	LOC151484	MYOD1
FFAR3	SLC27A2	ALDH3B1	BUB3
FLJ33630	MPI	MPHOSPH9	LOC100508624
PPP1R15B	C8orf4	C9orf68	MASP1
CACNG2	C6orf147	NMNAT2	SNX15
STOX1	SH2D3C	CCDC126	KIAA0495
CHRNA4	MYO7A	REXO1	DIS3L
WDFY3	C2orf65	BDKRB2	RARRES2
FLJ31715	LRSAM1	SSTR2	VENTX
ELFN1	SERPINA6	BTG4	RBBP4
GTF3C4	HS6ST3	CD36	LOC100130054
EIF4E	AURKC	APEH	KIF16B
LOC283508	RNF126P1	GGT2	SH2D4B

SLC46A1	C14orf162	LOC100129794	GALK2
AVIL	GSK3A	ARHGEF38	RPRD1A
DTWD2	ACADS	GDAP2	LYSMD2
LOC100507193	KHDRBS1	PRODH	DUT
GYS1	SOCS3	RPAIN	DISP2
TTLL9	C21orf93	UTP20	GRIN2C
INGX	MMD	MYBBP1A	SLC39A6
COX6B2	LOC255167	SLC41A2	MORN4
NCRNA00253	C10orf110	PRODH2	ZNF197
FLG2	LOC148413	LOC646014	RELL1
PHACTR2	PHF5A	LOC100288693	DUSP21
UBE2F	C12orf43	PACRG	FMR1NB
SYNJ2	LOC440900	SLC16A8	FLJ37543
TNXB	SLX4	NDUFA7	TRUB1
KIAA1383	PAAF1	LOC100130557	SCAND2
MDK	ALG3	LOC100508544	ZKSCAN3
PNCK	C1orf115	LRP5L	MPO
RMND5B	OR14J1	ENTPD7	LOC643072
KLHDC1	CIZ1	COX7B2	GPR45
GATA6	EPHA2	LOC283861	LOC728073
ZFHX2	C12orf67	RANBP3	STK17B
DCTN4	ETFDH	CYTH2	SCUBE2
LOC100287616	SEPT7P2	IREB2	SERF1A
FBXO34	ZNF718	LOC100506798	SERF1B
EYS	HSPA12B	TSHB	ADCY2
NPEPPS	SLC6A3	SNX3	MAN2B2
SNPH	TEX13B	PCSK7	NAGPA
LOC285819	LOC100129852	ACOT4	SREK1
PMPCA	ZNF239	PER2	C16orf3
SLC35D2	FAM101B	C7orf26	SEBOX
CDKN1A	CA1	PHYHD1	TP53BP1
EIF2AK2	CACNG1	DEFB106A	ARID3B
CPA6	SRL	DEFB106B	MDM1
TMEM63B	ALG2	SUPT7L	B4GALNT1
RFK	AK7	ADCY8	RPRD1B
PTRH1	ZNF213	VPS52	TTYH2
IFNGR1	CDH15	PRKRIR	TAS2R4
LOC100506958	SDS	HIGD1A	B4GALT3
SVEP1	FIZ1	KCNJ16	CNTNAP1
NAPA	TCEB3C	OXT	SMARCD1
CDON	GTPBP1	TUBGCP5	CYorf15A
LOC100506629	TMED7	PVRL2	RBM14
AP2A1	GSTCD	SLMO2	SLC30A9
LILRB4	BTF3L4	CDC14B	TARS2

PCOLCE2	GABBR2	REG4	C17orf60
FOXI1	TRYX3	HDC	ABLIM2
SHISA5	MRPS30	RGL3	POM121C
LAMA3	DPY19L1P1	FOXA2	PTPN21
LOC51152	FIBCD1	MT1P2	SLC38A7
PPP2R1A	KIAA1310	OR10H3	SGSH
REPIN1	SRGAP3	LOC100288447	PRO1483
PART1	SEC22C	HSF1	RBMS1
LOC283682	TTTY13	GDAP1	ADCK4
C11orf54	ZBTB39	BTNL9	STAMBPL1
CWF19L2	HSPA5	RPS28	CBX4
CCR3	HSP90AB1	DGAT1	PVR
TARBP2	SULF2	LOC541473	C16orf59
VAX2	GLYCTK	ST3GAL3	IL1RL1
KRTAP9-3	TMEM33	APOA1BP	LOC100133660
SIK1	DKFZp761P0212	C13orf18	TUSC5
RALGPS2	JUB	RAPGEF1	LOC728558
PTCHD2	CD163	LOC100505862	CCNDBP1
KEL	ZNF229	MYO15B	AKR1E2
SHISA4	FOXS1	PMAIP1	LOC100294362
EIF2S3	BBC3	ERLIN1	FLVCR2
COPS2	HCFC1	SEC23IP	WNT3
KCNAB3	SF3A1	EDF1	FOXL1
MYH7B	RABEP1	GTPBP5	ZZZ3
CEL	LOC100288911	KCNA7	GOLGA8C
LOC100508206	SLC8A3	AMELX	GOLGA8DP
RHOBTB2	LIN7A	RAD9A	GOLGA8E
KBTBD3	F11	PIN1P1	GOLGA8G
LOC100310756	RND3	SARM1	LOC653061
RBM23	MNT	TGOLN2	DNAJB12
WDR87	EDEM1	RG9MTD2	RBFOX1
NPTN	C19orf44	ANO1	UBE2N
LOC100506563	NLGN4X	C15orf32	C17orf46
DPY19L1	ACRBP	DNAH1	SNORA71B
TGM7	BEX1	GRWD1	ARMC4
HBM	ZNF222	C10orf72	NCRNA00213
C17orf65	NSMCE4A	DERA	PCDHB7
KLB	ELP3	PARD6G	PARP9
SDPR	LOC100132735	ZNF708	PROP1
CASKIN2	MAPK8IP1	PACRGL	SLC32A1
TMEM170B	LOC727818	C8B	LOC100130468
ACADSB	ATF2	KIR3DX1	LOC100129845
FAM196A	DNAJB13	TRIM21	SSX4
PRKD3	OR1J4	TIAM1	SSX4B

SRP19	KLK2	LARS2	SNRNP35
RNF133	ADH6	RBBP7	PLA2G4C
RNF41	LOC100510224	SULT1E1	CEACAM3
ERP29	RAB11A	FOLR3	C20orf118
LOC91316	GART	FLJ36665	MSTN
EDIL3	TMEM164	MADD	LOC100132051
HSPH1	YY1AP1	LEAP2	ST8SIA1
YEATS4	SLC29A2	LOC100128868	TRBV7-3
MKI67	OLFM4	CROT	FGD4
LOC285463	BANF2	CORO2A	MAGEB6
LOC100288590	LOC100129335	CYP2C19	ZNF613
SLAMF9	C9orf27	TMEM22	IL12RB1
PRKAB2	LOC642313	EGFEM1P	CYC1
CCDC42B	TAS2R3	LOC100131354	LGALS1
ANXA2P1	TSEN34	ZC3H7B	LOC100130987
RHBDD2	PPOX	CALM1	C7orf40
LOC283112	CASP9	LOC400931	LOC285949
FAM170B	LOC100287081	SPATS2L	DYNLL1
LOC400550	LOC100288701	LOC145783	VGF
SCD	EPB41L1	RFC1	ZDHHC1
LOC100508591	MDM4	RNF215	SRP14
GPR110	HTR7P1	ZNF157	LOC253842
DCAF4L2	ATL3	KCNMA1	ACY1
TSNARE1	RIMKLA	LOC100302650	COL11A1
UPK1B	CA12	LOC401317	PIK3R4
MSH3	ZNF202	C14orf167	SLFNL1
LOC100506216	NDUFB6	SPRYD4	EFCAB2
CACNG8	CCBE1	ZFYVE26	NFKBID
DSC1	FLJ35220	SNTG1	TBX2
CATSPERB	DOLK	LOC100509703	P4HB
PCYOX1L	FXYD2	IPO9	NT5M
ASH1L	MT1X	DAB2IP	LOC404266
LCE2B	GRIN3B	SLC17A9	TGFB1
LOC100288069	RAB28	PPFIA3	WASH7P
OS9	KCNK16	ANO4	MR1
TMLHE	ZNF417	KIAA0509	ATG9B
SGOL1	NPHP4	KRTAP4-6	CNOT4
PAX1	ARFGAP3	NDRG3	LOC100507950
ASS1	C20orf151	PRB3	RILP
EIF1AX	LRIG1	C9orf117	C8orf66
CELA2B	SLC9A9	ME3	TAAR1
PSG5	PDS5A	IRAK3	KIAA1632
LOC728196	TMEM191A	NEIL1	CAND1
PNKP	HRC	LRRC36	ATF7IP

KCNK1	IL11RA	PAGE1	TRIP10
GPR143	DDAH2	LOC100507291	STH
COL7A1	MBLAC1	GNG4	EP400NL
FBLN2	PEF1	TEKT1	SOX17
DEPDC7	ABHD14A	RNPEP	PKN3
NTAN1	EGLN2	C10orf113	ZNF498
IZUMO4	SCAMP4	LOC100289058	FLJ45825
ADCK2	PPP3R2	USP30	CABP4
USH1G	XIRP1	USP45	C7orf71
DMRTC1	LNP1	AFG3L1P	FOXH1
DMRTC1B	CASP8	TIRAP	OIP5
LOC100287598	C14orf82	NUCB1	ACLY
MKNK2	CPT1A	SLC25A48	TMEM85
C5orf20	SLC28A1	XKR6	IGLV6-57
ZNF175	D21S2088E	LOC286126	GRAP
HAND1	LOC116437	C3orf35	DUOX1
UNC13A	AP3B2	CARD11	C15orf57
SLCO2B1	NPC2	MYPN	APBB2
ZNF434	MFAP1	FAM123B	GDF15
BPESC1	N6AMT1	GTF2A1L	SDF4
AUH	SH3TC1	PSMF1	POU2F1
BAG3	CCDC97	ZNF625	C18orf49
LOC100507266	ZRANB3	LOC646762	C16orf13
ARL15	SAAL1	CNTNAP4	PARP6
KLRC4	MDS2	CD99P1	FAM170A
DHX35	C9orf116	LOC100287166	AGPAT4
OSCAR	DDX51	UBE2L6	KRT71
FAM47C	PTGER2	IGF1R	C18orf20
NKAIN1	ING5	LOC100133321	ZDHHC5
MRPL24	CCT2	LOC723809	SOX2OT
NKRF	TAT	GRIP1	C7orf43
EXPH5	FDXR	SCN1B	LOC644172
ZNF839	IGFBP1	UBQLN1	SLC15A4
PIGB	C3orf18	C12orf66	SNW1
KDM1A	HNRNPF	NAAA	SAE1
OGFR	ASGR2	TG	LOC399900
NDUFC1	TTC36	WDR25	B4GALT1
LOXL2	ISL2	C1QTNF4	ENSA
CUL9	NMNAT1	NOC2L	RUVBL1
C10orf99	SSC5D	ZNF211	CELF3
PLA2G3	GALK1	FOXK1	SCARNA2
REN	FAM178B	ANKS6	CD209
RUFY2	ELOVL5	FAM9C	ZNF518B
MGC57346	XCR1	AACSP1	LOC283693

ADRA1A	LOC100134713	HACL1	HAT1
IKBKG	LOC100287525	LOC285074	FNBP1L
SLC25A35	ENTHD1	C8orf30A	C1orf180
AIFM1	C1orf106	C1orf114	ZNF696
SESN3	SNX13	C21orf2	RBM45
C8orf60	C3orf33	SCN10A	ZNF232
AHSG	SIGLEC15	USP36	MKL1
PURB	EXOC1	TNXA	ATP5J
TUT1	CD1D	MUC5AC	SEC23B
SLN	HGFAC	STK32B	TPPP3
C17orf75	LOC100506021	ODF1	A2LD1
CALHM1	ATRN	PLAC8L1	ASXL2
LOC100509612	VPS33A	NCRNA00028	LOC115110
LILRB5	NUDC	KRT74	FGF21
SIRPG	MAT2A	LOC100507630	GHRL
PNPLA2	SPR	RPTOR	C1orf190
NXPH4	STRN4	HS3ST4	PRSS30P
HIST1H2AB	9-Sep	PTPRN2	NOX5
TAF6L	SLC12A4	SLC1A5	MAGEB2
SMPD3	NXF2	IFI35	AMIGO1
TMEM196	ORF1	PTK2B	ZNF7
MT1M	TCEB3B	SCARF2	MYH1
LOC100128292	CCDC11	FAM132B	HLA-A
C10orf46	LOC100508797	LAP3	CASQ1
MPP3	FAM66C	SF3B2	TMOD4
ABCF3	PHC3	ATP12A	C17orf70
TRIM28	ZNF75A	C12orf61	ACTL8
SSBP2	UNC50	PRKCE	DKKL1
AFF3	GPR150	TSLP	C1orf151
CNN3	ANKRD42	TMEM156	LOC121952
GBP4	PIP5K1A	SCARB1	CORIN
LOC284898	MAGOH2	FAM131A	IL6R
FLJ25917	LOC439911	FBF1	DBT
KIAA0226	ZMIZ2	APLP1	ATP13A4
C1orf134	NALCN	MRPL10	KDM6B
POLRMT	TRIM34	ZNF852	ATP6V1A
CNO	TRIM6-TRIM34	BAMBI	FECH
ZNF844	MAZ	GPR27	LOC284023
UFSP1	LOC285771	KLK11	DOCK10
PSPN	CSF1	BCORL1	LOC100288617
RHO	PCDHB17	SNRNP25	DOK6
IQCC	DENND1A	YAP1	ZNF493
GIPR	C9orf62	KLF4	QPCT
DNAI1	PPFIBP1	NKAIN2	LOC100129196

FAM83H
ATP6
ZNF628
NDP
TRIM31
F3
GJD2
LOC647979
CYP2A7
SCTR
C16orf93
NXN
DUSP10
RPL13AP5
RPL13AP6
FBXL3
MAVS
LOC100507109
GPR114
WNT4
NBEA
CHST8
LOC729420
NEDD4L
AKNA
MDH2
DPPA2
CETP
RIBC1
CXorf51
LOC100129034
ALDH1L1
CLCA3P
FCGR2C
HMGA2
CES4A
RBM22
KIAA1462
RBMX2
SCARB2
UBE2J2
ABCG1
C14orf159
COL20A1
UAP1L1
NUP62
CIB2
LRRC45
SLC43A2
ZNF442
FAH
PLK5P
HIST1H4C
CHMP4B
C1orf192
NPM2
DENR
PSAP
NSA2
FAM149B1
TTC30A
C6orf108
OR52A1
C1orf123
C3orf62
CEP57L1
FABP2
ATP5A1
NCRNA00119
RGS9
EPAG
S100A5
LOC144742
WFDC9
LOC90784
IL9
TSPAN6
STAU1
LOC400643
FBXL6
RBM11
JMJD8

Supplementary Table S2. Short-listed 1470 genes were co-expressed with SOX7 by Pearson correlation (FDR< 0.01) in GSE3744

PAPOLB	C16orf71	LOC100505942	C15orf60
SCN4A	FLJ21408	C1orf56	DDX25
ASB15	UTS2R	HCN2	LOC730091
LOC100507928	HECW1	LOC728537	CNTN4
WFDC12	HBD	LCN6	PKD2L1
LOC284100	SSTR3	GLE1	GHRHR
LOC730495	HTR6	CXorf36	LOC100289509
PDZD7	ATP4A	IL20RB	PAX2
PRLH	LOC100287024	TFR2	CALML3
NEK10	FBXO36	AMIGO3	FKBP4
CCDC157	NOS1AP	LIAS	ABCA9
TFAP2D	LOC285986	INCENP	TM7SF4
OR5P3	ZSCAN10	LOC100508426	LOC100287627
LOC100131532	LOC285281	CXorf22	TUBGCP2
LOC283868	DBP	WDR64	LOC653786
SLC47A1	CNDP2	LOC100506070	OTOA
NAT8B	LOC100289061	TUBB4Q	GRIA1
CBY3	LENEP	IGHV5-78	MDGA1
NAT8	TTR	CDHR3	FLJ35424
UBXN10	HAR1A	C14orf93	PHF7
C10orf62	KCNC2	ISM2	ACTL7B
SLC6A17	LOC100271832	LOC100509030	TXNDC8
POU5F2	SLC25A25	ANKMY1	TAS2R41
IGSF1	CALCA	LOC100133991	C11orf86
LOC100287576	CSTT	LOC157627	C3P1
TACR3	NCRNA00204B	ACCN4	NODAL
NKX1-1	PLUNC	C7orf69	LOC100289070
FAM166A	CCDC19	LDB3	CHGA
APOA4	HRH2	PDE1B	NCRNA00095
LOC100506319	CDRT15L2	PTHLH	RGAG1
LOC641364	ZFX	LOC100507040	C1QTNF6
CLPS	FLJ40292	TRY6	ZACN
ADAMTS13	C9orf29	TMEM132C	SLC45A1
LOC100129617	LOC100144602	ENG	RHOXF1
LOC253962	CNP	C17orf64	LOC284260
TSPY1	SPTBN4	NAIF1	TMEM88
SOX1	MGC16703	ST6GALNAC4	RNLS
NRL	MYCBPAP	PLA2G12B	GSTTP1
CYP2C9	LOC100287241	ETV1	CNGB3
HNRNPCL1	SUN5	CCDC40	KCNJ15
LOC440563	VIL1	NFS1	PPP1R2P9
LOC649330	TNRC18	GUCA1A	IL1F9

HIGD1B	GJC2	OGDHL	SERPINA7
TIMP4	LOC338667	SPHK2	MPP2
NHLH1	NCR2	LOC100505554	CCDC81
STARD8	MT4	ZCWPW1	FLJ36116
TCN1	KIR2DL1	SLC4A9	TRPA1
C6orf201	IL24	SLC4A4	DHX34
LRRC4B	AFM	GRIK1-AS	NPY5R
CA4	TTC9B	NCRNA00174	ISLR2
CLVS2	PPP5C	SFXN5	SLC5A5
LOC642891	C22orf43	TRIB1	SLC35D3
GRIK3	VEPH1	DDO	CARTPT
ZNF664	NEU2	MAPKAPK2	NCRNA00029
ADIG	C1orf46	PAPPA2	NR4A3
DEFB4A	CDK15	TRPM3	NPHS2
ASB16	LOC100506446	ARHGAP36	LOC90586
LMAN1L	COL4A6	FLT3	12-Sep
ZNF90	TES	DDX56	KLRC3
PRKACG	C7orf51	LOC100287483	SERPINA12
KLHL1	NEFH	LOC100287114	SAMD15
WDR17	CDH22	SPATA4	LOC647310
RRP12	SLC34A3	LOC100288490	SLC16A11
FHAD1	ABCA2	C6orf125	UBE3B
C10orf53	WFDC8	MAPK8IP2	KIAA0895L
C8A	GOLGA8IP	ATP6V0D2	BTF3
MGC23270	ZFYVE28	OSM	C1orf173
PTCRA	GNAT2	LEO1	PER1
FLT3LG	C12orf50	ICA1L	C22orf15
CPLX3	FAM155B	TAL1	NPBWR2
IL20	LOC221122	MEPE	CALHM3
NR2E3	HEXA	FLJ46026	CHST5
OGDH	TMEM159	VWCE	TMSB10
C3orf24	NKX6-3	PCSK1	RG9MTD3
CLN6	TP53TG5	MRGPRX4	SSH1
GNB5	LOC283859	LOC728606	CGB
MYH4	DOC2A	DEFB118	CGB5
NCRNA00118	ZMYND10	C9orf135	CGB7
C9orf44	LOC100505498	KIAA1045	MAPRE3
PA2G4P4	SFTPA1	CC2D2A	GHSR
LOC100130331	CCDC106	ZNF396	OR2H1
AQP2	IL4	LOC100287911	CYP27B1
RAB40A	VPS53	KBTBD10	NCRNA00184
LOC100129895	CACNA1I	RHAG	LOC100128977
DNAJC11	HIPK4	CPAMD8	LOC390940
TMEM151B	C10orf67	PRSS2	ZNF517

DDN	SNAP25	TBXA2R	TMEM144
STAT5B	LOC100130522	LOC100293160	C11orf76
RASGRP4	KCNK10	FAM71E1	TPM3
DNASE1	MYL2	ARID3A	DLEC1
TK2	LOC154092	SLC38A10	HOXA11-AS
YY2	FAM188B	C11orf35	RUNDC2A
MEGF11	ATF7	REPS2	IL1RAPL1
DCLK2	TMC2	SERPIND1	LOC100506797
RPL23AP53	LOC440104	SPACA3	DKFZp761E198
GSX2	RPL28	PLXNA2	EMX2OS
PRIC285	POMGNT1	WDR31	FLJ31356
ZRSR1	LOC100134317	LOC100291232	CTRB2
CROCCP3	LOC284412	PLEKHM1P	PDE12
PPIP5K1	LOC100506856	ATCAY	ARMCX4
GMPPB	KIAA1661	PLCH2	FAM83E
LOC100505515	RGS7	EGR4	ZG16
SPIRE2	RTF1	FAM55C	PDZD3
NEK9	LOC100506002	PRH1	C4orf17
LOC147791	CCDC85B	PRSS3	SLC6A18
NAF1	GNL1	FAM120AOS	CECR2
CCDC140	IQCH	SPAG6	ATP1A1OS
CPA1	C3orf15	LOC100133985	ETS2
MBTPS2	C10orf88	ANKS4B	UCN
ARF3	TEX13A	BAI2	C9orf9
HPS1	SCRN1	PDZK1	ACR
AK1	RUNDC1	CDHR5	LOC339666
TGM4	LOC100128262	NKD1	RFFL
EIF4G2	LOC100130691	NECAB2	GAL3ST3
PVT1	SSFA2	MOV10L1	ZNF527
PAX8	TXLNG	CYP4F2	KCNG3
LOC400927	CHP	HTR3C	LOC100129098
CYP1A2	C9orf131	HAP1	ITGAX
LOC100505820	C9orf144	CCDC85C	ARMC9
PLB1	FLJ41170	AMELY	MYH8
LOC440131	KCNJ5	KIAA1614	C3orf32
LOC100287728	SCGB1C1	LOC100329135	FGD2
VIPR2	ELOVL2	BCL6B	LOC100507198
HBE1	LOC731424	MAT1A	TAAR2
LOC100507164	ACTL6B	NBPF4	PRSS50
AK5	HNRNPM	ZNF331	ANKRD31
C10orf91	PROC	TAS2R39	KIAA1409
C5orf4	WDR76	MBD1	OLA1
FBXO39	FRMD8	CD300LG	BRPF3
FLT4	AMPD3	SSTR5	CACNA2D4

COASY	MRAP	FNDC8	KATNAL2
MEGF10	KIRREL2	MGC50722	RSPH3
TRAF7	TTC26	GAD2	HNRNPA1L2
LOC283761	SYT3	DCDC2B	CES1P1
NUDT10	EML2	CD24	PAH
DUSP26	TPP1	LAMC3	RABGGTA
FAM22F	RNF122	GLOD5	PTPRT
SLC2A9	SP5	BAI1	CACNA2D3
ATP2A2	COX4I2	CCR10	RDH8
RPS27	LRRN3	WNT16	C20orf181
RIMBP3	GNAT3	ONECUT1	VHL
RIMBP3B	TSPAN16	SLC22A9	MPV17L
RIMBP3C	LRP1B	ZNF445	TAPBP
CPB1	HUWE1	LRFN1	LOC646168
EIF4EBP2	SLC30A3	THOP1	LOC100131733
SCG3	DNASE2B	ANKRD29	TMEM201
ZNF79	LOC100509661	FSD2	GAMT
CHRM2	OVOL3	CPNE4	SORCS2
GAPVD1	ARHGEF15	DSCR4	ADAD1
FAM55D	ASMT	LASS1	MUC17
POLD2	MBOAT7	C17orf88	TNFSF9
FAM24A	LOC100132207	PPP6R1	LCN10
TPD52L3	ABO	ART1	SCN3B
PEAR1	HNRNPA1P10	FAM41AY1	C5AR1
ADRB3	CYGB	FAM41AY2	LOC729683
C1orf27	SP8	C1orf91	BPIL2
ZNF490	MPPED1	LOC151171	GPER
SCARF1	GATC	GRN	UBE2O
SAMD14	SLC5A10	CCIN	ZNF174
ANKRD23	AGFG2	CD34	DSPP
RDM1	WASH5P	FAM162B	ZNF17
PIGG	HGC6.3	INSL3	PLAC4
ZBTB16	HEATR4	PPP1R1C	ATP6V0A1
ATOH8	LOC153682	ATP9B	C18orf25
CDHR2	LOC441601	CRH	ZNF598
CIB3	MBL1P	ADAMTS7	ZNF843
TSGA13	TPH1	CCL15	WDTC1
TMEM221	ENOX1	UGP2	GLS2
C20orf166	C7orf63	TSFM	AAGAB
LOC100133445	DNAJA4	LAGE3	CCDC96
PPM1N	C16orf74	LOC100287704	SCLY
CELA3B	C9orf103	HNRNPC	CYorf15B
ZFY	UQCC	LOC79999	FOLH1B
DGCR11	TAS2R38	RDH5	FLJ11292

KIF24	CRYBB1	WDR78	CHGB
FLRT1	LOC100132062	ZNF736	NCOA5
PROCR	LOC100133161	CALM3	AIRE
ITPK1	LOC731275	KCNIP3	TOLLIP
LOC340094	LOC100129268	SLC12A9	LY6G6F
RGS12	RFX2	TRIM66	FLYWCH1
LOC400768	SEC14L2	ASB14	NCRNA00260
BRI3BP	ASTN2	C17orf68	OPN1LW
11-Mar	TBC1D28	GARNL3	OPN1MW
LOC100507250	DYDC2	LOC100133669	OPN1MW2
NR1H3	TOR1A	ZNF324B	ANKRD2
SLC2A8	SLC6A13	CH25H	SIX5
NRSN1	C12orf65	HBEGF	SCN8A
RNF157	ACP5	C14orf104	TESC
ACTR3C	LOC100133308	NHLH2	ZER1
KCNC4	SDSL	NFE2L2	SUV420H2
TM9SF4	LOC339807	KCND3	SPATA2
FAM76A	CNTD2	RTN1	SMC1A
PRO2964	C6orf165	RUNDC3A	LOC100132078
CAV3	SALL4	NNT	NFASC
DEFA6	IGSF22	ZNF781	LOC643714
CNR1	FAM122A	ADPRHL1	DYSFIP1
EPHX2	ADAMTSL4	NOX1	LRRC33
GNS	LOC283174	GMPR	CYMP
C19orf30	LOC220980	LOC100508853	WDR1
LOC100507066	PI4K2A	SLC22A8	COMMD4
LOC100507384	LOC100233209	C16orf92	RCE1
MED13L	LOC100133920	CSPP1	CACNB2
SLC9A3	ZNF135	DYRK3	SERTAD2
LOC1720	LOC100287813	ADD1	PHRF1
FBXL18	SEMA6D	KANK3	LOC339166
REM2	LOC100505470	LPIN3	LOC284939
LOC728012	LOC646576	SULT2B1	MMD2
NXNL2	RADIL	SLC26A3	SNX27
MYH15	FCRLB	PDE4C	STAC3
RBP2	SLC27A5	LOC100507408	NOL9
HNRPA1P5	ZNF81	PADI3	SULT4A1
LOC642817	TMEM120B	NLGN4Y	KCNK17
TLN2	GALNT5	RTN4IP1	BMP8A
FOXD2	UBQLN3	DPF1	SNED1
VPS18	LENG9	FAM182A	GPX8
TEX11	OR51M1	FZR1	MTHFR
LOC100128416	HPS4	ZC3H12A	DKFZp566F0947
ARHGAP6	APOOL	KLHL25	ZNF219

C7orf50	DUX2	SCAMP5	RASSF9
FUZ	SRSF4	SARDH	NKX2-8
LOC100509011	NCAM2	GOLGA6L4	EPO
PRSS47	DUX4	LTB4R	FLJ32955
GGT7	DUX4L2	POU3F1	PRMT8
C2orf81	DUX4L3	FLJ14107	LOC100129461
PA2G4	DUX4L4	L1TD1	FGF10
TRPM5	DUX4L5	PRG4	LOC283484
RBBP8	DUX4L6	DUOXA1	SDK2
LCE1E	DUX4L7	LOC339400	GPR62
AP4S1	C9orf79	LOC283403	PTGIR
MUC3A	NR6A1	MERTK	MNS1
DUSP1	TSGA14	C4orf23	DPPA3
ADAMTS4	NNAT	SPN	TEX101
ADCY1	MGC14436	SRC	LOC100129726
SREBF1	SFTPB	LOC100130264	LOC100506553
TSPYL1	CAPN11	FAM165B	SYTL5
FAM102A	DDX3X	GLTSCR1	ABR
HIVEP3	LOC100287556	EGR2	LOC253805
ABCB9	RECQL5	MYOC	LRFN3
LOC387723	STAB2	LOC100505555	HIST1H1T
FOXJ1	C20orf132	TLR5	ACSM3
LOC222159	C1orf55	FLJ12334	BASE
LOC100288123	HACE1	PRB1	DHDDS
TEK	OTOR	EGR3	PPIAL4A
C19orf29	GCNT4	CCT6B	LCN8
LRRC46	FCN2	C20orf26	LOC613266
C11orf72	ACP2	NTS	EPDR1
SCN9A	RNF208	CATSPER2P1	THTPA
TUBG1	ENPP7	HYAL3	OPCML
LOC642980	G6PC3	CASC4	LOC445341
FLJ32063	LOC100507255	ZNF528	HCG27
VAV2	CXorf64	C12orf42	GOLGA6L1
LOC285638	SEMA4A	CPNE6	GOLGA6L6
LOC415056	DCAF11	MED25	HSFX1
ARL17B	EHD4	ZSWIM5	HSFX2
LOC100130654	NDUFA10	LOC100506080	NECAB3
GAD1	ADAMTS10	KIF9	WRAP53
TSNAXIP1	EHBP1L1	PNMAL2	SETDB1
FCGR2A	PAX4	FASN	ANKFY1
RRAGB	PMS1	CLIP3	AOC2
SIK3	ZNF254	GRINA	LOC100129716
KIR2DS1	GLRB	MTOR	C16orf81
PIGO	FAM154A	PARD3B	CELF5

SEPN1	C18orf2	ZNF556	C4orf43
LOC100507371	FLJ40288	NCRNA00167	LOC253044
CASP2	SHE	ERGIC3	SKA1
PRDM14	LOC389765	NCRNA00265	MYO3B
SDHB	FOXRED2	DDX53	KCNRG
XDH	LOC145837	PROM2	SPINK13
LOC100130938	FKSG2	CAMK2N2	ZC3HAV1L
KLHL22	DBF4B	SNORD8	SEMA5B
SIRT3	UNC93A	EIF2AK4	B4GALNT2
RAB40AL	PSME3	RLTPR	C11orf65
SYK	PQLC2	ATE1	ZNF846
SOAT2	HS3ST6	DEFA5	MAP3K12
APOB48R	SPATA18	SPG11	HOXD9
LILRA1	LOC100130856	TOR2A	LOC644714
TPPP	GRIN3A	FAM82A2	TRIM25
NUP210	HLCS	PRPH2	ADC
LOC100129503	TMCO5B	TMEM175	NPY1R
RAD21L1	SRRT	FOSB	SP2
PTGES	C12orf34	EVI5	LONRF2
C10orf112	TCTN2	COL13A1	HOXA4
C21orf84	LOC644090	LOC729652	ADM2
PMCHL2	TMEM136	XGPY2	UTP15
LOC284385	SPRR2G	FAM46B	PHF21B
LOC100132726	NCL	LOC339988	CHM
LOC100506819	LOC100505644	SYN2	LOC728099
GPR144	BTRC	RDH13	C21orf74
IGSF9B	ROBO4	LOC100507454	SYCE1
KIR2DS2	ANKRD39	LOC100289333	APOL5
ATRIP	PRCD	GGN	ZNF385A
GOLGA6B	SPAM1	ZNF585A	FBXO15
GOLGA6D	IPO4	CCDC57	LOC100134445
CD33	CCDC103	PLEKHM1	PTGER1
AMAC1	C1orf49	HTR1B	HTR2C
SV2A	WEE1	OPTC	RUNDC2C
FREM1	PRPF19	ARPP19	LOC286154
ZC3H3	HSPA4	SNORA43	PEX19
LOC100287590	PRDX3	INHBC	ASCC3
C11orf36	MAGEB3	ASAP1	LOC100287529
SERPINB13	FNDC1	OVOL1	LOC388889
DMBX1	C4orf37	CHIA	TSPYL6
LOC284788	LOC84931	UPK3B	RPS14
ZNF876P	SLC5A8	C7orf53	PDIA2
PRKACB	TCTN1	FAM120A	FAM100A
RGS5	MGC45922	KCNH6	DKFZP434L187

SLC22A11	SERPINA4	LOC100132273	KLK13
LRRC6	NOLC1	LOC100510391	C17orf53
FAT4	SPAG8	HFE	LOC100505711
KLHL4	SEMA3F	MAPK13	C5orf45
LOC100287045	CST11	RABL3	KCNA4
OR2B6	UNQ6975	SEZ6L	AHCYL2
ZNF75D	CDX1	MAP6	PRR12
RIPPLY2	FLJ35934	LOC646982	C10orf103
OPA3	SIGLEC6	HDAC8	LOC339593
SLC18A2	ESR1	TTBK1	LOC100288985
AGTR1	LOC284551	C17orf55	KIF12
C1orf84	BAHCC1	ROBO2	SIAH2
PAK7	RFX6	LOC283788	TREML1
ADRBK1	XAGE3	ULBP3	GPRIN3
CPT2	SFTPD	KCNT1	THBD
KCNJ9	FSIP1	MS4A5	CD68
THAP6	LOC286442	DNAH3	TTTY5
FAM167B	PIGQ	LOC286297	YSK4
TRPM4	ANP32C	MOBP	KCNJ6
CXorf27	PSMD4	ALDH16A1	ZNF597
DOCK1	TDP1	LOC26172	APOM
NCOR2	LOC100131642	PTGER3	ALDOB
VPS26B	LOC100506267	LOC390705	LOC400541
LOC388387	C1orf105	LOC100506880	CD80
KRT31	LOC100507443	PYGO2	SLC37A2
TRAF3IP1	FAM49A	MYH16	ZNF366
KCTD17	HIPK2	OK/SW-CL.36	FRMPD4
NUDCD3	LOC100129463	TUBGCP4	IL1F7
BSDC1	OR2C1	MEGF8	SHROOM1
NADK	KRTAP5-9	LOC100216545	LOC100499221
SPACA1	NAALADL1	LOC100289045	POFUT2
LOC100509093	NUP62CL	MANEAL	TRIM9
NCSTN	LOC100286999	UCN2	PRUNE
RIN1	FAM116B	HBB	CAMK1
ZNF808	MFAP3	ZGLP1	WWOX
FAM160A1	GLI4	ACSM2B	DSTNP2
MST1P2	ELAVL4	PDIA3	CSE1L
BTD	GPR120	CBLN3	TMX4
SEZ6L2	PARP10	SLC43A1	LGALS3BP
ZNF589	GAS2L2	CHRNB2	CALML6
LOC100507816	APOC3	C4orf6	AXIN2
LOC286254	GFRA4	SNRPF	LOC100506848
TREML2P1	GPIHBP1	PRRG2	GTF2A2
DOCK5	ASPG	ARHGEF11	ULBP1

LOC100506728	AIP
PHF20L1	VCX2
KIAA1239	LOC100169752
TMEM150A	OR1G1
AGXT	C1orf89
NAPSA	CDNF
SCN2B	PIH1D1
ALPPL2	MPZL2
LOC440602	CCDC117
FAM138A	C15orf17
FAM138B	MYOZ3
FAM138C	HAUS1
FAM138E	ATP6AP1
FAM138F	LOC283070
LOC152578	UNC45B
LOC100506677	OR9A1P
C6orf1	TSSK2
C19orf57	GYPA
RASGRF2	PPM1M
MOCS1	DCDC5
PIGT	MTERFD3
LOC728804	KLRC1
IQCF5	KLRC2
LOC400238	MAG
ELFN2	TTPAL
C9orf98	TSSK4
GFM2	
HIBADH	
FAT1	
LOC100287482	
FLOT1	
INPP5A	
IGSF8	
TRANK1	
CABLES2	
ATPAF1	
IMPDH2	
LOC100129722	
NTRK2	
ZNF655	
PLK3	
FMO6P	
UBTF	
CDR1	

Supplementary Table S3

Name	ID	Text	PubMed IDs	Concept ID	DOID
**SOX4**	6659	indings suggest that Sox4 contributes to the malignant phenotype of adenoid cystic carcinoma cells by promoting cell survival	16636670	C0006826	462
**SOX4**	6659	indings suggest that Sox4 contributes to the malignant phenotype of adenoid cystic carcinoma cells by promoting cell survival	16636670	C0010606	4866
**SOX4**	6659	The human transcription factor SOX4 was 5-fold up-regulated in bladder tumors compared with normal tissue based on whole-genome expression profiling of 166 clinical bladder tumor samples and 27 normal urothelium samples.	16585165	C0005695	5428
**SOX4 - Ortho**	20677	Data indicate that Sox4 and 17 can act as both antagonists and agonists of beta-catenin/TCF activity, and this mechanism may regulate Wnt signaling responses in many developmental and disease contexts.	17875931	C0012634	4
**SOX17**	64321	SOX17 silencing due to promoter hypermethylation is an early event during tumorigenesis and may contribute to aberrant activation of Wnt signaling in colorectal cancer.	18413743	C0009402	1994
**SOX17 - Ortho**	20671	Data indicate that Sox4 and 17 can act as both antagonists and agonists of beta-catenin/TCF activity, and this mechanism may regulate Wnt signaling responses in many developmental and disease contexts.	17875931	C0012634	4
**SMAD3**	4088	A surface hydrophobic corridor within the MH2 domain of Smad3 is critical for association with CAN/Nup214 and nuclear import; Smad3 and Smad4 have different susceptibility to inhibition of import by cytoplasmic retention factor SARA	12917407	C0034494	11260
**SMAD3**	4088	c-Jun NH(2)-terminal kinase tended to induce the phosphorylation of Smad2/3L in human colorectal adenoma-carcinoma sequence.	15665291	C0001430	657
**SMAD3**	4088	examined the possible deterioration in the pathway in human squamous cancer cell lines, focusing on intracellular localization of S100C/A11 and its functional partners Smad3 and Smad4-	17476473	C0006826	462
**SMAD3**	4088	examined the possible deterioration in the pathway in human squamous cancer cell lines, focusing on intracellular localization of S100C/A11 and its functional partners Smad3 and Smad4-	17476473	C0936282	4766
**SMAD3**	4088	increased TGF-beta-Smad signaling in sporadic and familial ALS and impaired TGF-beta signal transduction in neurons of sporadic ALS patients, presumably at the step of pSmad2/3 translocation into the nucleus	18210139	C0002736	332
**SMAD3**	4088	increased TGF-beta-Smad signaling in sporadic and familial ALS and impaired TGF-beta signal transduction in neurons of sporadic ALS patients, presumably at the step of pSmad2/3 translocation into the nucleus	18210139	C0002736	332
**SMAD3**	4088	interactions between AR, Smad3, and Smad4 may result in the differential regulation of the AR transactivation, which further strengthens their roles in the prostate cancer progression	12226080	C0376358	10283
**SMAD3**	4088	Observational study of gene-disease association and gene-gene interaction. (HuGE Navigator)	18095154	C0012634	4

**SMAD3**	4088	Observational study of gene-disease association. (HuGE Navigator)	14727154	C0012634	4
**SMAD3**	4088	Observational study of gene-disease association. (HuGE Navigator)	16886151	C0012634	4
**SMAD3**	4088	Observational study of gene-disease association. (HuGE Navigator)	18445023	C0012634	4
**SMAD3**	4088	Protein and mRNA levels of SMAD3, but not of SMAD4 or SMAD7, were variably elevated in scleroderma fibroblasts	12847691	C0011644	419
**SMAD3**	4088	Protein and mRNA levels of SMAD3, but not of SMAD4 or SMAD7, were variably elevated in scleroderma fibroblasts	12847691	C0036421	418
**SMAD3**	4088	Smad3 gene mutations could be associated with the pathogenesis of human osteoarthritis	12939660	C0029408	455
**SMAD3**	4088	Smad3 is unlikely to function as a classical tumor suppressor gene in the pathogenesis of sporadic parathyroid or enteropancreatic endocrine tumors.	12161532	C0027651	162
**SMAD3**	4088	Smad3, through regulating angiogenic molecule expression in tumor cells, is critical for progression of prostate cancer	17908958	C0376358	10283
**SMAD3**	4088	TGF-beta1 inhibited IFN-gamma and TNF-alpha-induced TARC production in HaCaT cells via Smad2/3. Modulation of TGF-beta/Smad signaling pathway may be beneficial for treatment of atopic dermatitis.	12615364	C0011615	3310
**SMAD3**	4088	TGF-beta(1) regulates proliferation through Smad3 signaling in both fibroblast populations; however, it is the levels of HA generated by the cells that influence the outcome of this response.	18174158	C0019338	10883
**SMAD3**	4088	TGF-beta(1) regulates proliferation through Smad3 signaling in both fibroblast populations; however, it is the levels of HA generated by the cells that influence the outcome of this response.	18174158	C0020505	8669
**SMAD3**	4088	TGF-beta-mediated activation of the ALK5-Smad 3 pathway plays a role in SHH promoted motility and invasiveness of gastric cancer cells	18174246	C0024623	10534
**SMAD3 - Ortho**	17127	Smad3, through regulating angiogenic molecule expression in tumor cells, is critical for progression of prostate cancer	17908958	C0376358	10283
**SMAD7**	4092	Gene variants may weakly contribute to a particular genetic background that increases the susceptibility to development of type 2 diabetes.	17931948	C0011860	9352
**SMAD7**	4092	In Helicobacter pylori-infected gastric mucosa, interferon-gamma induces expression of Smad7, which then prevents endogenous TGF-beta 1 from down-regulating ongoing tissue-damaging Th1 response.	14988821	C0079487	3686
**SMAD7**	4092	Observational study of gene-disease association. (HuGE Navigator)	18231913	C0012634	4
**SMAD7**	4092	Observational study of gene-disease association. (HuGE Navigator)	18445023	C0012634	4
**SMAD7**	4092	results suggest that alterations in the Smad pathway, including marked Smad7 deficiency and Smad3 up-regulation, may be responsible for TGF-beta hyperresponsiveness observed in scleroderma	11904440	C0011644	419
**SMAD7**	4092	results suggest that alterations in the Smad pathway, including marked Smad7 deficiency and Smad3 up-regulation, may be responsible for TGF-beta hyperresponsiveness observed in scleroderma	11904440	C0036421	418
**SMAD7**	4092	Single nucleotide polymorphism in SMAD7 is associated with	17934461	C0009402	1994

		colorectal cancer			
**SMAD7**	4092	Smad7 and Smurf1 have roles in regulation of TGF-beta signaling in scleroderma fibroblasts	14722617	C0011644	419
**SMAD7**	4092	Smad7 and Smurf1 have roles in regulation of TGF-beta signaling in scleroderma fibroblasts	14722617	C0036421	418
**SMAD7**	4092	Smad7 appears to be upregulated in endometrial cancers compared to normal endometrium.	15661223	C0476089	2871
**SMAD7**	4092	Smad7 was overexpressed in ARO anaplastic cell line, the most malignant follicular thyroid carcinoma.	12952364	C0006826	462
**SMAD7**	4092	Stable overexpression of Smad7 in human melanoma cells impairs bone metastasis.	17332363	C0025202	1909
**SMAD7**	4092	Stable overexpression of Smad7 in human melanoma cells impairs bone metastasis.	17332363	C0153690	2759
**SMAD7**	4092	TGFbeta rapidly induces nuclear translocation of Smad proteins and subsequently stimulates Smad-Sp1 complex formation which increases Sp1 binding to promoter boxes in a pancreatic cancer cell line.	16714330	C0346647	1793
**SMAD7**	4092	variation at SMAD7 does not significantly contribute to an inherited susceptibility to CLL	18231913	C0023458	1040
**SMAD7**	4092	Various cellular functions implicated in melanoma development may be under control of autocrine TGF-beta and may be inhibited by Smad7 expression	16007121	C0025202	1909
**CTNNB1**	1499	Aberrant accumulation of beta-catenin is very common in parathyroid tumors, and is caused by stabilizing homozygous mutation in 7.3% of Swedish patients.	18541010	C0030521	11006
**CTNNB1**	1499	Aberrant beta-catenin expression may play an important role in the histologic differentiation and tumor staging of mucoepidermoid carcinoma.	12694354	C0206694	4531
**CTNNB1**	1499	a beta-catenin-RET kinase pathway is a critical contributor to the development and metastasis of thyroid carcinoma	18316596	C0027627	2619
**CTNNB1**	1499	a beta-catenin-RET kinase pathway is a critical contributor to the development and metastasis of thyroid carcinoma	18316596	C0549473	3963
**CTNNB1**	1499	Abnormal beta-catenin gene expression with invasiveness of primary hepatocellular carcinoma in China.	11819825	C0019204	684
**CTNNB1**	1499	Abnormal E-cadherin and alpha-catenin and beta-catenin in pancreatic carcinoma tissues. Abnormal E-cadherin and alpha-catenin with differentiation, lymph node and liver metastases. Aberrant beta-catenin with lymph node and liver metastases.	12532469	C0235974	4905
**CTNNB1**	1499	Abnormal E-cadherin and alpha-catenin and beta-catenin in pancreatic carcinoma tissues. Abnormal E-cadherin and alpha-catenin with differentiation, lymph node and liver metastases. Aberrant beta-catenin with lymph node and liver metastases.	12532469	C0494165	12722
**CTNNB1**	1499	abnormal expression of beta-catenin in gastric carcinoma and survival	12452049	C0699791	5517
**CTNNB1**	1499	abnormal immunhistochemical E-cadherin and beta-catenin expression is associated with changes in pit pattern in invasive colorectal neoplasms	15289833	C0009404	1985
**CTNNB1**	1499	accumulation of beta-catenin in colorectal cancer is related to chromosomal instability	17203186	C0009402	1994
**CTNNB1**	1499	All 61 meningothelial meningiomas, 10 of 12 invasive meningiomas, and 3 of 5 anaplastic meningiomas were positive for both ECAD and beta-catenin, while these were both negative in all of the fibrous meningiomas.	15696778	C0025286	3565
**CTNNB1**	1499	All 61 meningothelial meningiomas, 10 of 12 invasive	15696778	C0259785	5613

		meningiomas, and 3 of 5 anaplastic meningiomas were positive for both ECAD and beta-catenin, while these were both negative in all of the fibrous meningiomas.			
**CTNNB1**	1499	All 61 meningothelial meningiomas, 10 of 12 invasive meningiomas, and 3 of 5 anaplastic meningiomas were positive for both ECAD and beta-catenin, while these were both negative in all of the fibrous meningiomas.	15696778	C0334605	7212
**CTNNB1**	1499	All 61 meningothelial meningiomas, 10 of 12 invasive meningiomas, and 3 of 5 anaplastic meningiomas were positive for both ECAD and beta-catenin, while these were both negative in all of the fibrous meningiomas.	15696778	C0334606	7211
**CTNNB1**	1499	A loss of nuclear beta-catenin is the most consistent feature in prostate cancer rather than absolute levels of expression	18459111	C0376358	10283
**CTNNB1**	1499	Alterations in adenomatous polyposis coli/beta-catenin pathway and cyclin D1 dysregulation may contribute to pathogenesis of pleuropulmonary desmoid tumors and solitary fibrous tumors.	17090192	C1266119	5955
**CTNNB1**	1499	Altered beta-catenin distribution in gastric cancer may result from the imbalance of E-cadherin production and Wnt expression, which confers on gastric cancer cells more aggressive behaviors.	16132582	C0024623	10534
**CTNNB1**	1499	Altered beta-catenin distribution in gastric cancer may result from the imbalance of E-cadherin production and Wnt expression, which confers on gastric cancer cells more aggressive behaviors.	16132582	C0024623	10534
**CTNNB1**	1499	analysis of Epstein-Barr virus, beta-catenin, and E-cadherin in gastric carcinomas	17982235	C0699791	5517
**CTNNB1**	1499	an internally truncated LRP5 receptor is strongly implicated in deregulated activation of the WNT/beta-catenin signaling pathway in hyperparathyroid tumors	18044981	C0027651	162
**CTNNB1**	1499	Anticancer-drug-induced apoptotic cell death in leukemia cells is associated with proteolysis of beta-catenin. beta-Catenin plays a role in promoting Jurkat survival.	12127563	C0023418	1240
**CTNNB1**	1499	beta-catenin accumulates in the nucleus of epithelial cells of juvenile polyps	15557107	C0221273	7111
**CTNNB1**	1499	Beta-catenin gene mutations are a peculiar feature of skin tumors with matrical differentiation and correlate with a pattern of intense and diffuse beta-catenin nuclear expression.	14633602	C0037286	3165
**CTNNB1**	1499	beta-catenin has a role in progression of colorectal neoplasms	14977843	C0009404	1985
**CTNNB1**	1499	Beta catenin induced human melanoma growth requires the downstream target Microphthalmia-associated transcription factor.	12235125	C0025202	1909
**CTNNB1**	1499	Beta-catenin may have an important role in the development of malignancy and in the determination of biological features of keratoacanthoma and squamous cell carcinoma of the skin.	17679465	C0006826	462
**CTNNB1**	1499	Beta-catenin may have an important role in the development of malignancy and in the determination of biological features of keratoacanthoma and squamous cell carcinoma of the skin.	17679465	C0007137	1749
**CTNNB1**	1499	Beta-catenin may have an important role in the development of malignancy and in the determination of biological features of keratoacanthoma and squamous cell carcinoma of the skin.	17679465	C0936282	4766
**CTNNB1**	1499	beta-catenin may have a role in progression of colorectal neoplasms to metastatic liver lesions	17168211	C0009404	1985
**CTNNB1**	1499	Beta-catenin might be related to the occurrence and development of kidney tumor.	11831984	C0022665	263
**CTNNB1**	1499	beta-catenin mutation and its nuclear localization are frequent causes of Wnt signaling pathway activation suggesting that	16378715	C0206711	5374

		beta-catenin activation mutations contribute to tumorigenesis of pilomatricomas			
**CTNNB1**	1499	beta-catenin nuclear accumulation plays a role in Dukes' D human colorectal cancers	15254684	C0009402	1994
**CTNNB1**	1499	beta-catenin, p53 and PCNA may play important roles in the carcinogenesis of colorectal adenoma.	12515622	C0001430	657
**CTNNB1**	1499	beta-catenin plays a central role in mesenchymal cells during the healing process, and is an appealing therapeutic target for disorders of wound healing.	15654359	C0012634	4
**CTNNB1**	1499	beta-catenin plays an important role in oncogenesis through the crossregulation of NF-kappa B in breast and colonic neoplasms	12398896	C0009375	235
**CTNNB1**	1499	beta-catenin plays a role in endometrial carcinogenesis, particularly in endometrioid carcinomas	11957146	C0206687	3000
**CTNNB1**	1499	beta-catenin signalling is essential in sustaining the cancer stem cell phenotype	18385740	C0006826	462
**CTNNB1**	1499	beta-catenin signalling is essential in sustaining the cancer stem cell phenotype	18385740	C0936282	4766
**CTNNB1**	1499	Beta-catenin simultaneously induces activation of the p53-p21WAF1 pathway and overexpression of cyclin D1 during tumor cell differentiation	15111320	C0027651	162
**CTNNB1**	1499	Beta-catenin stabilization because of either beta-catenin or AXIN I mutation might be a late event for malignant progression rather than an early genetic event involving the initiation of HCC development.	15698401	C0006826	462
**CTNNB1**	1499	beta-catenin/TCF transcriptional activity is blocked by Cdx1 and Cdx2, which then inhibits colon cancer cell proliferation	15215241	C0007102	219
**CTNNB1**	1499	Bilateral Wilms tumours showed loss of the wild type WT1 allele (loss of heterozygosity (LOH)) and a tumour specific mutation in catenin beta1 (CTNNB1).	17551084	C0027651	162
**CTNNB1**	1499	Bilateral Wilms tumours showed loss of the wild type WT1 allele (loss of heterozygosity (LOH)) and a tumour specific mutation in catenin beta1 (CTNNB1).	17551084	C0027708	2154
**CTNNB1**	1499	Bilateral Wilms tumours showed loss of the wild type WT1 allele (loss of heterozygosity (LOH)) and a tumour specific mutation in catenin beta1 (CTNNB1).	17551084	C0281267	6741
**CTNNB1**	1499	colon cancer cells retain significant amounts of LEF-1 induced nuclear beta-catenin compared to LEF-1 transfected normal epithelial cells; beta-Catenin binds directly to CRM1 & overexpression of CRM1 reduces nuclear beta-catenin-mediated transactivation	16378739	C0007102	219
**CTNNB1**	1499	Combined increased p53 and reduced membranous beta-catenin protein expression indicated a very poor prognosis in patients with esophageal squamous cell carcinoma.	18455580	C0279626	3748
**CTNNB1**	1499	Correlation between beta-catenin mutations and expression of Wnt-signaling target genes in hepatocellular carcinoma.	18282277	C0019204	684
**CTNNB1**	1499	COX-2 and beta-Catenin may have roles in regulating intracellular Survivin levels in mouse and human colon cancer	17128412	C0007102	219
**CTNNB1**	1499	CTNNB1 mutations were found in 2/19 adenomas without APC mutation.	12737446	C0001430	657
**CTNNB1**	1499	CTNNB1 signaling plays a critical role in the development of a significant fraction of prostate cancers.	11921277	C0376358	10283
**CTNNB1**	1499	CTNNB1 was expressed strongly on the cuboidal cell membranes and cytoplasm in both tumor types, but to a lesser extent in the polygonal cells, localizing mainly in the cytoplasm.	17383052	C0027651	162

**CTNNB1**	1499	data indicate that altered expression of beta-catenin may play an important role in oral cancer progression through increased proliferation and invasiveness under epidermal growth factor receptor (EGFR) activation but not mutation or cyclin D1 expression	15791567	C0153381	8894
**CTNNB1**	1499	Data indicate that Sox4 and 17 can act as both antagonists and agonists of beta-catenin/TCF activity, and this mechanism may regulate Wnt signaling responses in many developmental and disease contexts.	17875931	C0012634	4
**CTNNB1**	1499	Data show that beta-catenin is overexpressed in Kaposi sarcoma and primary effusion lymphoma, and that this overexpression is regulated by the Kaposi's sarcoma-associated herpesvirus (KSHV) latency-associated nuclear antigen LANA.	12592400	C0036220	8632
**CTNNB1**	1499	Data show that beta-catenin is overexpressed in Kaposi sarcoma and primary effusion lymphoma, and that this overexpression is regulated by the Kaposi's sarcoma-associated herpesvirus (KSHV) latency-associated nuclear antigen LANA.	12592400	C0036220	8632
**CTNNB1**	1499	Data suggest a beta-catenin-dependent, stage-specific role for Notch1 signaling in promoting the progression of primary melanoma.	16239965	C0025202	1909
**CTNNB1**	1499	Data suggest the Wnt/beta-catenin signaling pathway may be associated with ethanol-induced osteonecrosis.	18288545	C0520474	10159
**CTNNB1**	1499	Decreased beta-catenin expression is associated with advanced gallbladder cancers	17341890	C0153452	8090
**CTNNB1**	1499	Decreased immunoexpression of beta-catenin and E-cadherin in serous ovarian tumors may be helpful in identifying the cases of higher metastatic potential and infiltration ability.	18655368	C0919267	2394
**CTNNB1**	1499	Deregulated beta-catenin signaling may occur in high-risk neuroblastomas without MYCN amplification resulting in transactivation of MYC and other target genes to induce an aggressive phenotype.	17724465	C0027819	769
**CTNNB1**	1499	dysregulation of beta-catenin may contribute to pancreatic duct adenocarcinoma progression through distinct mechanisms	12679314	C0001418	299
**CTNNB1**	1499	dysregulation of beta-catenin may contribute to pancreatic duct adenocarcinoma progression through distinct mechanisms	12679314	C0281361	4074
**CTNNB1**	1499	dysregulation of beta-catenin may contribute to pancreatic duct adenocarcinoma progression through distinct mechanisms	12679314	C1176475	3007
**CTNNB1**	1499	E-cadherin and beta-catenin have roles in progression of Epstein-Barr virus-associated gastric carcinoma	17663505	C0007097	305
**CTNNB1**	1499	Elevated expression of cell-cycle regulators p16(INK4A), p21(CIP1), and cytoplasmic/nuclear beta-catenin correlated with increased colorectal cancers risk, as did elevated expression of survivin and human telomerase reverse transcriptase.	17641414	C0009402	1994
**CTNNB1**	1499	Endogenous KIT and beta-catenin were found to associate in mast cell leukemia cells, and in vitro kinase assay demonstrated that active KIT phosphorylates tyrosine residues of beta-catenin directly.	17949810	C0023461	9254
**CTNNB1**	1499	Epigenetic repression of DACT3 leads to aberrant Wnt-beta-catenin signaling in colorectal cancer cells.	18538736	C0009402	1994
**CTNNB1**	1499	ErbB-beta-catenin complexes are associated with human infiltrating ductal breast and murine mammary tumor virus (MMTV)-Wnt-1 and MMTV-c-Neu transgenic carcinomas	11950845	C0007097	305
**CTNNB1**	1499	Exposure of colon cancer cells to nitric oxide unraveled a so-far-unidentified mechanism of beta-catenin regulation.	17030184	C0007102	219

**CTNNB1**	1499	Expression and prognostic roles of beta-catenin in hepatocellular carcinoma correlation with tumor progression and postoperative survival	11839663	C0019204	684
**CTNNB1**	1499	Expression of e-cadherin and beta-catenin in human esophageal squamous cell carcinoma relationships with prognosis.	12532436	C0279626	3748
**CTNNB1**	1499	Fascin is a novel target of beta-catenin-TCF signaling and is expressed at the invasive front of human colon cancer	17638895	C0007102	219
**CTNNB1**	1499	FGF9 mutant tumors showed normal membranous beta-catenin expression and the absence of mutation in the beta-catenin gene	18165946	C0027651	162
**CTNNB1**	1499	findings indicate that alterations of beta-catenin are frequent in cancer of the uterine cervix and suggest that they may play an important role in the development of these tumors	11893906	C0006826	462
**CTNNB1**	1499	findings indicate that alterations of beta-catenin are frequent in cancer of the uterine cervix and suggest that they may play an important role in the development of these tumors	11893906	C0027651	162
**CTNNB1**	1499	findings indicate that alterations of beta-catenin are frequent in cancer of the uterine cervix and suggest that they may play an important role in the development of these tumors	11893906	C0936282	4766
**CTNNB1**	1499	findings show that WTX, a protein encoded by a gene mutated in Wilms tumors, forms a complex with beta-catenin, AXIN1, beta-TrCP2 and APC; data provide a possible mechanistic explanation for the tumor suppressor activity of WTX	17510365	C0027708	2154
**CTNNB1**	1499	Findings suggest that phospho-beta-catenin accumulation in Alzheimer's disease might result from impaired proteasome function.	15781969	C0002395	10652
**CTNNB1**	1499	functional significance of combined dysregulation of PKD1 and E-cadherin in prostate cancer; their effect on cell growth is mediated by beta-catenin.	17979146	C0376358	10283
**CTNNB1**	1499	GSK3beta-dependent protein degradation was switched between Hath1 and beta-catenin by Wnt signaling, leading to the dramatic alteration of cell status between proliferation and differentiation in colon cancer	17241872	C0007102	219
**CTNNB1**	1499	hepatocellular carcinomas (HCC) with extensive methylation harbor frequent beta-catenin mutations; HCCs with high levels of chromosomal instability are associated with p53 mutations, suggesting presence of two independent pathways for pathogenesis of HCC	17510384	C0019204	684
**CTNNB1**	1499	HER-2/neu transcriptionally activates Jab1 expression via the AKT/beta-catenin pathway in breast cancer cells.	17914096	C0006142	4241
**CTNNB1**	1499	High nuclear expression of beta-catenin is correlated with locally advanced colorectal cancer	15126105	C0009402	1994
**CTNNB1**	1499	High Pin1 expression in primary prostate cancer markedly inhibits the beta-catenin interaction with androgen receptor.	16428447	C0376358	10283
**CTNNB1**	1499	immunohistochemistry for b-catenin may be a powerful tool for detecting those adenomas with an increased risk for malinancy.	17903198	C0001430	657
**CTNNB1**	1499	Immunohistochemistry of cyclin D1 and beta-catenin, and mutational analysis of exon 3 of beta-catenin gene in parathyroid adenomas	11836555	C0262587	7608
**CTNNB1**	1499	Immunohistological examination of nuclear accumulation of beta-catenin may be useful for diagnosing malignant immunohistological examination of nuclear accumulation of beta-catenin may thus be useful for diagnosing malignant PLTs.	16465411	C0006826	462
**CTNNB1**	1499	in breast and lung tumor cells, MDA-7 protein expression	12907143	C0024121	3683

		modulates cell-cell adhesion and intracellular signaling via coordinate regulation of the beta-catenin and PI3K pathways			
**CTNNB1**	1499	increased expression predicts favorable prognosis in resected nonsmall cell lung carcinoma	11857309	C0684249	3905
**CTNNB1**	1499	In esophageal adenocarcinomas, nuclear translocation of beta-catenin was observed regardless of the expression of APC.	16407829	C0279628	4914
**CTNNB1**	1499	inhibition of beta-catenin and in effect c-myc expression through activation of PPARgamma may help prostate cancer cells to restore several characteristics of normal prostate cells phenotype	17466258	C0376358	10283
**CTNNB1**	1499	In HT29 and HCT116 colorectal cancer cells, beta-catenin/TCF transcriptional activity is inhibited by AP-2alpha due to formation of AP-2alpha/APC/beta-catenin complex.	15331612	C0009402	1994
**CTNNB1**	1499	In human pilomatricoma, the frequency of beta-catenin gene mutations was remarkably low (30%),in Exon 3 of the beta-catenin gene .	12575848	C0206711	5374
**CTNNB1**	1499	Interaction of MUCI with CTNNB modulates cyclin D1 in H. pylori-induced stomach neoplasms.	17393422	C0038356	4713
**CTNNB1**	1499	Loss of beta-catenin expression is a strong and independent predictor of an unfavorable outcome in patients with endometrial carcinoma.	17504383	C0476089	2871
**CTNNB1**	1499	Loss of beta-catenin may result in the disruption of the function of the cell-cell adhesion complex, which may cause weak cell-cell adhesion and confer invasive properties on a tumor.	11747475	C0027651	162
**CTNNB1**	1499	Loss of expression of E-cadherin and beta-catenin may play an important role in the progression of pulmonary adenocarcinoma.	15660698	C0152013	3910
**CTNNB1**	1499	Lower levels of nuclear beta-catenin is associated with prostate cancer progression	15455387	C0376358	10283
**CTNNB1**	1499	Lysophosphatidic acid induced colon cancer cell proliferation requires the beta-catenin signaling pathway.	15837931	C0007102	219
**CTNNB1**	1499	matrix metalloproteinase-2 and 9 and membrane-type 1 matrix metalloproteinase mRNA expression in endometriosis was higher than in normal endometrium whereas E-cadherin, alpha- and beta-catenin mRNA expression was not suppressed in endometriosis	12587534	C0014175	289
**CTNNB1**	1499	membranous overexpression of E-cadherin and beta-catenin are associated with the metastatic prostate cancer cells in bone and the high frequency of expression suggests their involvement in the intercellular adhesion of the metastatic cells in bone	18008331	C0346957	2332
**CTNNB1**	1499	membranous overexpression of E-cadherin and beta-catenin are associated with the metastatic prostate cancer cells in bone and the high frequency of expression suggests their involvement in the intercellular adhesion of the metastatic cells in bone	18008331	C0376358	10283
**CTNNB1**	1499	methylation may play important role in progression and metastasis of small bowel carcinoid tumors	17526942	C0027627	2619
**CTNNB1**	1499	Molecular genetic analysis of malignant melanomas for aberrations of the WNT signaling pathway genes CTNNB1, APC, ICAT and BTRC.	12124804	C0025202	1909
**CTNNB1**	1499	MUC1 may affect cancer cell migration by increasing E-cadherin/beta-catenin complex formation and restoring E-cadherin membrane localization	17764657	C0006826	462
**CTNNB1**	1499	MUC1 may affect cancer cell migration by increasing E-cadherin/beta-catenin complex formation and restoring E-	17764657	C0936282	4766

		cadherin membrane localization			
**CTNNB1**	1499	Mutation in exon 3 of the beta-catenin gene was found in 2 of the 20 endometrial cancer samples; however, it was not found in the 25 endometrial hyperplasias or the 20 associated hyperplasias.	12111402	C0006826	462
**CTNNB1**	1499	Mutation in exon 3 of the beta-catenin gene was found in 2 of the 20 endometrial cancer samples; however, it was not found in the 25 endometrial hyperplasias or the 20 associated hyperplasias.	12111402	C0007103	5654
**CTNNB1**	1499	Mutation in exon 3 of the beta-catenin gene was found in 2 of the 20 endometrial cancer samples; however, it was not found in the 25 endometrial hyperplasias or the 20 associated hyperplasias.	12111402	C0014173	8835
**CTNNB1**	1499	Mutation in exon 3 of the beta-catenin gene was found in 2 of the 20 endometrial cancer samples; however, it was not found in the 25 endometrial hyperplasias or the 20 associated hyperplasias.	12111402	C0476089	2871
**CTNNB1**	1499	Mutation in exon 3 of the beta-catenin gene was found in 2 of the 20 endometrial cancer samples; however, it was not found in the 25 endometrial hyperplasias or the 20 associated hyperplasias.	12111402	C0936282	4766
**CTNNB1**	1499	Mutations in CTNNB1 are associated with melanoma	18384130	C0025202	1909
**CTNNB1**	1499	Mutations of CTNNB1 may not be a factor in tumorigenesis of cervical cancer	12883680	C0007847	4361
**CTNNB1**	1499	Mutations of CTNNB1 were observed in cases of gastric cancer.	18330950	C0024623	10534
**CTNNB1**	1499	mutations rare in ulcerative colitis-related colorectal carcinomas	11920497	C0009324	8577
**CTNNB1**	1499	negative immunoreactivity of beta-catenin in serous carcinomas and the presence of residual tumor seem to be useful markers in selecting patients likely to have an unfavorable course	15381903	C0206701	3114
**CTNNB1**	1499	No evidence of mutations in parathyroid adenomas.	17284619	C0262587	7608
**CTNNB1**	1499	Nuclear beta-catenin expression correlating with the grade of IEN in polyps and carcinomas supports its role in colorectal carcinogenesis.	18330089	C0007097	305
**CTNNB1**	1499	nuclear beta-catenin expression significantly related to ulcerative growth of colorectal cancer	11953860	C0009402	1994
**CTNNB1**	1499	Nuclear beta-catenin expression was significantly greater in Multiple colorectal adenoma patients' tumours than in sporadic adenomas.	17505512	C0001430	657
**CTNNB1**	1499	Nuclear beta-catenin expression was significantly greater in Multiple colorectal adenoma patients' tumours than in sporadic adenomas.	17505512	C0001430	657
**CTNNB1**	1499	nuclear beta-catenin immunostaining can serve as a sensitive immunohistochemical marker for the diagnosis of endometrial stromal tumors	18376386	C0334695	5166
**CTNNB1**	1499	Nuclear localization of beta-catenin, an indirect evidence of deregulated Wnt signaling pathway, was observed in 5 (19%) small intestinal adenocarcinomas and 36 (71%) colorectal adenocarcinomas.	16501564	C0278803	4906
**CTNNB1**	1499	Observational study of gene-disease association, gene-environment interaction, and pharmacogenomic / toxicogenomic. (HuGE Navigator)	18500270	C0012634	4
**CTNNB1**	1499	Observational study of gene-disease association. (HuGE Navigator)	15520370	C0012634	4

**CTNNB1**	1499	Observational study of gene-disease association. (HuGE Navigator)	15523694	C0012634	4
**CTNNB1**	1499	Observational study of gene-disease association. (HuGE Navigator)	16356174	C0012634	4
**CTNNB1**	1499	Observational study of gene-disease association. (HuGE Navigator)	16843107	C0012634	4
**CTNNB1**	1499	Observational study of gene-disease association. (HuGE Navigator)	17160944	C0012634	4
**CTNNB1**	1499	Observational study of gene-disease association. (HuGE Navigator)	18618575	C0012634	4
**CTNNB1**	1499	Observational study of gene-disease association. (HuGE Navigator)	18676680	C0012634	4
**CTNNB1**	1499	One out of 62 melanoma cell lines was found to carry a mutation in exon 3 of the beta-catenin gene indicating that aberration of the Wnt-1/wingless pathway through activation of beta-catenin is a rare event	11930117	C0025202	1909
**CTNNB1**	1499	our data indicate that inactivation of beta-catenin by a 3p21.3 homozygous deletion might be a crucial event in the development of the mesothelioma NCI-H28 cells.	12970740	C0025500	2645
**CTNNB1**	1499	Overexpression of beta-catenin is associated with hepatocellular carcinoma	12969793	C0019204	684
**CTNNB1**	1499	Phospho-beta-catenin may have a different involvement in invasive breast carcinomas, according to its subcellular distribution.	16474376	C0678222	3459
**CTNNB1**	1499	provides support that E-cadherin induction by WNT/beta-catenin signaling is an evolutionarily conserved pathway operative in lung cancer cells and that loss of expression may be important in lung cancer development or progression	12937339	C0006826	462
**CTNNB1**	1499	provides support that E-cadherin induction by WNT/beta-catenin signaling is an evolutionarily conserved pathway operative in lung cancer cells and that loss of expression may be important in lung cancer development or progression	12937339	C0242379	1324
**CTNNB1**	1499	provides support that E-cadherin induction by WNT/beta-catenin signaling is an evolutionarily conserved pathway operative in lung cancer cells and that loss of expression may be important in lung cancer development or progression	12937339	C0242379	1324
**CTNNB1**	1499	provides support that E-cadherin induction by WNT/beta-catenin signaling is an evolutionarily conserved pathway operative in lung cancer cells and that loss of expression may be important in lung cancer development or progression	12937339	C0936282	4766
**CTNNB1**	1499	PS/gamma-secretase-mediated cleavage of LAR controls LAR-beta-catenin interaction, suggesting an essential role for PS/gamma-secretase in the regulation of LAR signaling	17259169	C0036421	418
**CTNNB1**	1499	PS/gamma-secretase-mediated cleavage of LAR controls LAR-beta-catenin interaction, suggesting an essential role for PS/gamma-secretase in the regulation of LAR signaling	17259169	C0036421	418
**CTNNB1**	1499	PTPRK influences transactivating activity of beta-catenin in non-tumoral and neoplastic cells by regulating the balance between signaling and adhesive beta-catenin, thus providing biochemical basis for the hypothesis of PTPRK as a tumor suppressor gene.	18276111	C0027651	162
**CTNNB1**	1499	Reduced membranous expression of beta-catenin was associated with metastasis in salivary adenoid cystic carcinoma	16865250	C0010606	4866
**CTNNB1**	1499	Reduced membranous expression of beta-catenin was associated with metastasis in salivary adenoid cystic carcinoma	16865250	C0027627	2619

**CTNNB1**	1499	Regulation of leukemic cell adhesion, proliferation, and survival by beta-catenin.	12130512	C0023418	1240
**CTNNB1**	1499	Restoration of E-cadherin/beta-catenin expression in pancreatic cancer cells inhibits growth by induction of apoptosis.	12219004	C0346647	1793
**CTNNB1**	1499	results suggest an established Wnt signaling pathway in most gastric cancers, a close correlation of beta-catenin/TCF4-mediated signaling with tumor dissemination, and the unlikelihood of a direct effect of activated Wnt signaling on CD44 expression	16311123	C0024623	10534
**CTNNB1**	1499	results suggest an established Wnt signaling pathway in most gastric cancers, a close correlation of beta-catenin/TCF4-mediated signaling with tumor dissemination, and the unlikelihood of a direct effect of activated Wnt signaling on CD44 expression	16311123	C0027651	162
**CTNNB1**	1499	results suggest that the Wnt/beta-catenin signaling pathway plays dual functions in head and neck squamous cell carcinoma (HNSCC) development promoting both cell survival and invasive growth of HNSCC cells.	16084063	C0279671	3744
**CTNNB1**	1499	REVIEW of studies pointing to molecular mechanisms that govern the integration between cell-cell adhesion and gene expression, as reflected in the switches between these two functions of beta-catenin in colon cancer cells	17854061	C0007102	219
**CTNNB1**	1499	Role of Wnt pathway in medulloblastoma oncogenesis accumulation of beta-catenin in tumor cells was immunohistochemically proven in 5 cases; 2 cases showed positive immunoreactivity for Wnt-1 and another 2 showed mutation of either CTNNB1 or AXIN1	12209999	C0025149	3858
**CTNNB1**	1499	shRNAs targeted against beta-catenin could have a gene silencing effect and block the WNT signaling pathway. They could inhibit cell growth, increase apoptosis, and induce cell cycle arrest in human colon cancer cell lines.	18161931	C0007102	219
**CTNNB1**	1499	SOX2 and beta-catenin act in synergy in the transcription regulation of CCND1 in breast cancer cells	18456656	C0006142	4241
**CTNNB1**	1499	Splice variants of CTNNB1 ands downstream targets were used as markers for neoplastic progression of esophageal cancer.	16114033	C0014859	5041
**CTNNB1**	1499	Splice variants of CTNNB1 ands downstream targets were used as markers for neoplastic progression of esophageal cancer.	16114033	C0546837	1104
**CTNNB1**	1499	study examined the presence of mutations in TP53 at codon 249 (Ser-249, considered as a hallmark of mutagenesis by aflatoxin) and in CTNNB1 in circulating free DNA of patients with hepatocellular carcinoma or chronic liver disease from Alexandria, Egypt	18313840	C0019204	684
**CTNNB1**	1499	study found that Wnt/beta-catenin pathway is involved in the genesis of pulmonary sclerosing haemangioma (PSH), but mutation of exon 3 of the beta-catenin gene rarely contributes to the activation of the Wnt/beta-catenin pathway in PSH	17693578	C0002991	4418
**CTNNB1**	1499	study of correlation between mutations & expression of E-cadherin, beta-catenin, occludin & claudin & complexity of colon carcinoma growth; perturbed expression & distribution of these proteins was found, but could not be linked to complexity of growth	18397460	C0007097	305
**CTNNB1**	1499	study of correlation between mutations & expression of E-cadherin, beta-catenin, occludin & claudin & complexity of colon carcinoma growth; perturbed expression & distribution of these proteins was found, but could not be linked to complexity of growth	18397460	C0699790	1520

**CTNNB1**	1499	Suggest beta-catenin deregulation is involved in sporadic hepatoblastoma and also suggests that mismatch repair defects and p53 mutations contribute to this rare liver cancer.	17962810	C0206624	687
**CTNNB1**	1499	Suggest beta-catenin deregulation is involved in sporadic hepatoblastoma and also suggests that mismatch repair defects and p53 mutations contribute to this rare liver cancer.	17962810	C0345904	12300
**CTNNB1**	1499	TCF4 expression mediated by beta-catenin/p300 may be important for initial steps during trans-differentiation of endometrial carcinoma cells.	15806138	C0476089	2871
**CTNNB1**	1499	TGF-beta is a modulator of beta-Catenin levels in tumoral fibroblasts and non-tumoral fibroblasts, despite the oncogenic mutations already present in this gene in tumoral fibroblasts of desmoid tumors.	17601559	C0027651	162
**CTNNB1**	1499	TGF-beta is a modulator of beta-Catenin levels in tumoral fibroblasts and non-tumoral fibroblasts, despite the oncogenic mutations already present in this gene in tumoral fibroblasts of desmoid tumors.	17601559	C0027651	162
**CTNNB1**	1499	The aberrant expression of beta-catenin protein was statistically correlated to the lymph node metastasis in esophageal cancer.	12478897	C0014859	5041
**CTNNB1**	1499	The aberrant expression of beta-catenin protein was statistically correlated to the lymph node metastasis in esophageal cancer.	12478897	C0546837	1104
**CTNNB1**	1499	The aberrant expression of beta-catenin protein was statistically correlated to the lymph node metastasis in esophageal cancer.	12478897	C0686619	10437
**CTNNB1**	1499	The beta-catenin expression in hepatocellular carcinoma cells was heterogeneous among types of hepatitis viral infection	15832407	C0019204	684
**CTNNB1**	1499	the cross talk of KLF4 and beta-catenin plays a critical role in homeostasis of the normal intestine as well as in tumorigenesis of colorectal cancers.	16507986	C0009402	1994
**CTNNB1**	1499	The data suggest a novel role for tyrosine phosphorylation of N-cadherin by Src family kinases in the regulation of beta-catenin association during transendothelial migration of melanoma cells.	16371504	C0025202	1909
**CTNNB1**	1499	the expression manner of PTEN, beta-catenin, and p53 immunocytochemistry was observed in the normal endometrium (proliferative, secretory, and atrophic, and endometrial glandular and stromal breakdown[beta-catenin]	18335551	C0151514	2733
**CTNNB1**	1499	The MUC1/beta-catenin interaction occurs in primary tumors, & is dramatically increased in metastatic lesions.	12618757	C0677930	8176
**CTNNB1**	1499	the pathway including S100A7/psoriasin and beta-catenin signaling has a role in tumor progression of squamous cell carcinoma of oral cavity	18223693	C0007137	1749
**CTNNB1**	1499	The positive inter-regulation between beta-cat/Tcf-4 signaling and ET-1 signaling potentiates proliferation and survival of prostate cancer (CaP) cells, thereby representing a novel mechanism that contributes to CaP progression.	16291872	C0376358	10283
**CTNNB1**	1499	there are somatic mutations of APC & beta-catenin genes in desmoid-type fibromatosis & abnormalities in Wnt signal pathway; these abnormalities may result in aberrant cell proliferation & apoptosis, which may be important in tumorigenesis & progression	17558858	C0000768	759
**CTNNB1**	1499	there are somatic mutations of APC & beta-catenin genes in desmoid-type fibromatosis & abnormalities in Wnt signal pathway; these abnormalities may result in aberrant cell proliferation & apoptosis, which may be important in tumorigenesis & progression	17558858	C0000768	759

**CTNNB1**	1499	There is a possible role of progesterone in regulation of beta-catenin expression in endometrial tumors. Nuclear beta-catenin accumulation, like gene abnormalities, is associated with the alteration of tumor morphology due to progesterone.	12708483	C0000768	759
**CTNNB1**	1499	There is a possible role of progesterone in regulation of beta-catenin expression in endometrial tumors. Nuclear beta-catenin accumulation, like gene abnormalities, is associated with the alteration of tumor morphology due to progesterone.	12708483	C0474809	3001
**CTNNB1**	1499	These data indicate that somatic mutations affecting APC and CTNNB1 do not play a major role in the pathogenesis of sporadic ependymomas.	16843107	C0014474	4844
**CTNNB1**	1499	These data reveal a potentially important role for transcriptionally active beta-catenin in the regulation of Rad6B gene expression, and link aberrant beta-catenin signaling with transcriptional deregulation of Rad6B and breast cancer development.	17050667	C0006142	4241
**CTNNB1**	1499	These data reveal a potentially important role for transcriptionally active beta-catenin in the regulation of Rad6B gene expression, and link aberrant beta-catenin signaling with transcriptional deregulation of Rad6B and breast cancer development.	17050667	C0006826	462
**CTNNB1**	1499	These data reveal a potentially important role for transcriptionally active beta-catenin in the regulation of Rad6B gene expression, and link aberrant beta-catenin signaling with transcriptional deregulation of Rad6B and breast cancer development.	17050667	C0936282	4766
**CTNNB1**	1499	These data show activation of the Wnt/beta-catenin-signalling pathway in uveal melanoma and suggest that components of this pathway might be useful prognostic markers as well as attractive therapeutic targets to treat this disease.	17992121	C0012634	4
**CTNNB1**	1499	These data show activation of the Wnt/beta-catenin-signalling pathway in uveal melanoma and suggest that components of this pathway might be useful prognostic markers as well as attractive therapeutic targets to treat this disease.	17992121	C0220633	6039
**CTNNB1**	1499	These experiments suggest that in breast cancer cells, the expression of ZBP1 and the expression of beta-catenin are coordinately regulated.	18490442	C0006142	4241
**CTNNB1**	1499	These results indicate that the altered expression of beta-catenin, but not cyclin D1, in hepatocellular carcinoma may play an important role in tumor progression by stimulating tumor cell proliferation.	12692418	C0019204	684
**CTNNB1**	1499	These results unravel a novel pathway in the control of beta-catenin cellular transport and strongly suggest that SYT-SSX2 contributes to tumor development, in part through beta-catenin signaling	16462762	C0027651	162
**CTNNB1**	1499	The Wnt/beta-catenin signaling pathway may have a role in megakaryocytopoiesis in polycythemia vera and essential thrombocythemia.	18619646	C0040028	2224
**CTNNB1**	1499	This paper focuses on changes in E-cadherin (CDH1), adenomatous polyposis coli (APC), and beta-catenin (CTNNB1) in 50 tumors of the central nervous system	17905526	C0027651	162
**CTNNB1**	1499	Transendothelial migration is compromised in melanoma cells expressing a dominant-negative form of beta-catenin, thus supporting a regulatory role of beta-catenin signaling in this process.	15987741	C0025202	1909
**CTNNB1**	1499	Translocalization of beta-catenin is associated with invasion in gastric cancer	15138556	C0024623	10534
**CTNNB1**	1499	Tumor sections frmo colorectal cancer patients showed	15888491	C0027651	162

		elevated expression levels of AKT1, correlating with enhanced cytoplasmic/nuclear expression of beta-catenin.			
**CTNNB1**	1499	variations in beta-catenin protein levels were dependent on post-transcriptional mechanisms involving the Wnt/beta-catenin pathway only in leukemic cells	16688229	C0023418	1240
**CTNNB1**	1499	Vpu leads to the depression of both total and beta-catenin-associated E-cadherin levels through beta-TrCP-dependent stabilization of the transcriptional repressor Snail.	18256147	C0011570	1596
**CTNNB1**	1499	Vpu leads to the depression of both total and beta-catenin-associated E-cadherin levels through beta-TrCP-dependent stabilization of the transcriptional repressor Snail.	18256147	C0011581	1596
**CTNNB1**	1499	We show that a fraction of N-cadherin in a complex with catenins is associated with cholesterol/sphingolipid-rich membrane microdomains in aggressive melanoma cells in vitro and experimental melanomas in vivo.	17668445	C0025202	1909
**CTNNB1**	1499	We show that a fraction of N-cadherin in a complex with catenins is associated with cholesterol/sphingolipid-rich membrane microdomains in aggressive melanoma cells in vitro and experimental melanomas in vivo.	17668445	C0025205	4357
**CTNNB1**	1499	When colon cancer cells with high beta-catenin levels were treated with beta-catenin antisense ODNs, VEGF-A expression was reduced by more than 50%. There is a close link between beta-catenin signaling & VEGF-A expression regulation in colon cancer.	12810642	C0007102	219
**CTNNB1**	1499	When colon cancer cells with high beta-catenin levels were treated with beta-catenin antisense ODNs, VEGF-A expression was reduced by more than 50%. There is a close link between beta-catenin signaling & VEGF-A expression regulation in colon cancer.	12810642	C0007102	219
**CTNNB1**	1499	Wnt/beta-catenin activation was observed in 65% of pancreatic adenocarcinomas, independently of beta-catenin gene mutations in most tumors	16756720	C0027651	162
**CTNNB1**	1499	Wnt/beta-catenin activation was observed in 65% of pancreatic adenocarcinomas, independently of beta-catenin gene mutations in most tumors	16756720	C0281361	4074
**CTNNB1**	1499	Wnt/beta-catenin pathyway is activated by epigenetic inactivation of Dkk3, thereby promoting the growth of lung cancer cells	18048388	C0242379	1324
**CTNNB1**	1499	Wnt/beta-catenin signaling has a role in neurodevelopment as well as in neurodegenerative diseases [review]	17143299	C0524851	4874
**CTNNB1**	1499	Wnt/beta-catenin signalling pathway is activated in most of gastric cancers, which may play pivotal roles either in gastric cancer formation or in tumour invasion and dissemination	15896469	C0024623	10534
**CTNNB1**	1499	Wnt/beta-catenin signalling pathway is activated in most of gastric cancers, which may play pivotal roles either in gastric cancer formation or in tumour invasion and dissemination	15896469	C0024623	10534
**CTNNB1**	1499	Wnt signaling pathway associated with beta-catenin regulation in breast cancer tissue	15082903	C0006142	4241
**CTNNB1**	1499	Wnt signaling pathway associated with beta-catenin regulation in breast cancer tissue	15082903	C0006826	462
**CTNNB1**	1499	Wnt signaling pathway associated with beta-catenin regulation in breast cancer tissue	15082903	C0936282	4766
**CTNNB1**	1499	WT1 and WTX mutations occur with similar frequency, that they partially overlap in Wilms tumors, and that mutations in WT1, WTX, and CTNNB1 underlie the genetic basis of about one-third of Wilms tumors	18311776	C0027708	2154

**CTNNB1**	1499	WT1 and WTX mutations occur with similar frequency, that they partially overlap in Wilms tumors, and that mutations in WT1, WTX, and CTNNB1 underlie the genetic basis of about one-third of Wilms tumors	18311776	C0027708	2154
**CTNNB1 - Ortho**	12387	a beta-catenin-RET kinase pathway is a critical contributor to the development and metastasis of thyroid carcinoma	18316596	C0027627	2619
**CTNNB1 - Ortho**	12387	a beta-catenin-RET kinase pathway is a critical contributor to the development and metastasis of thyroid carcinoma	18316596	C0549473	3963
**CTNNB1 - Ortho**	12387	an internally truncated LRP5 receptor is strongly implicated in deregulated activation of the WNT/beta-catenin signaling pathway in hyperparathyroid tumors	18044981	C0027651	162
**CTNNB1 - Ortho**	12387	beta-catenin signalling is essential in sustaining the cancer stem cell phenotype	18385740	C0006826	462
**CTNNB1 - Ortho**	12387	beta-catenin signalling is essential in sustaining the cancer stem cell phenotype	18385740	C0936282	4766
**CTNNB1 - Ortho**	12387	the pathway including S100A7/psoriasin and beta-catenin signaling has a role in tumor progression of squamous cell carcinoma of oral cavity	18223693	C0007137	1749
**SNAIL1**	6615	The identification of a beta-catenin-T-cell factor-regulated Axin2-GSK3beta-Snail1 axis provides new mechanistic insights into cancer-associated epithelial-mesenchymal transition programmes.	17072303	C0006826	462
**SNAIL1**	6615	The identification of a beta-catenin-T-cell factor-regulated Axin2-GSK3beta-Snail1 axis provides new mechanistic insights into cancer-associated epithelial-mesenchymal transition programmes.	17072303	C0936282	4766
**WNT1**	7471	The identification of a beta-catenin-T-cell factor-regulated Axin2-GSK3beta-Snail1 axis provides new mechanistic insights into cancer-associated epithelial-mesenchymal transition programmes.	17072303	C0006826	462
**WNT1**	7471	The identification of a beta-catenin-T-cell factor-regulated Axin2-GSK3beta-Snail1 axis provides new mechanistic insights into cancer-associated epithelial-mesenchymal transition programmes.	17072303	C0936282	4766
**BTRC**	8945	a high level of p53 downregulates the beta-catenin expression, but this effect is attenuated by non-functional AXIN2 or betaTrCP in lung cancer.	18372914	C0242379	1324
